# Interplay between membrane active host defense peptides and heme modulates their assemblies and in vitro activity

**DOI:** 10.1038/s41598-021-97779-2

**Published:** 2021-09-15

**Authors:** Tünde Juhász, Mayra Quemé-Peña, Bence Kővágó, Judith Mihály, Maria Ricci, Kata Horváti, Szilvia Bősze, Ferenc Zsila, Tamás Beke-Somfai

**Affiliations:** 1 Institute of Materials and Environmental Chemistry, Research Centre for Natural Sciences, Budapest, Hungary; 2 Hevesy György PhD School of Chemistry, Eötvös Loránd University, Budapest, Hungary; 3 ELKH-ELTE Research Group of Peptide Chemistry, Eötvös Loránd University, Budapest, Hungary; 4 Department of Organic Chemistry, Eötvös Loránd University, Budapest, Hungary

**Keywords:** Biophysics, Chemical biology, Structural biology, Chemistry

## Abstract

In the emerging era of antimicrobial resistance, the susceptibility to co-infections of patients suffering from either acquired or inherited hemolytic disorders can lead to dramatic increase in mortality rates. Closely related, heme liberated during hemolysis is one of the major sources of iron, which is vital for both host and invading microorganisms. While recent intensive research in the field has demonstrated that heme exerts diverse local effects including impairment of immune cells functions, it is almost completely unknown how it may compromise key molecules of our innate immune system, such as antimicrobial host defense peptides (HDPs). Since HDPs hold great promise as natural therapeutic agents against antibiotic-resistant microbes, understanding the effects that may modulate their action in microbial infection is crucial. Here we explore how hemin can interact directly with selected HDPs and influence their structure and membrane activity. It is revealed that induced helical folding, large assembly formation, and altered membrane activity is promoted by hemin. However, these effects showed variations depending mainly on peptide selectivity toward charged lipids, and the affinity of the peptide and hemin to lipid bilayers. Hemin-peptide complexes are sought to form semi-folded co-assemblies, which are present even with model membranes resembling mammalian or bacterial lipid compositions. In vitro cell-based toxicity assays supported that toxic effects of HDPs could be attenuated due to their assembly formation. These results are in line with our previous findings on peptide-lipid-small molecule systems suggesting that small molecules present in the complex in vivo milieu can regulate HDP function. Inversely, diverse effects of endogenous compounds could also be manipulated by HDPs.

## Introduction

Hemolysis is a common symptom for patients suffering from malaria, bacterial sepsis, or from related genetic disorders such as sickle cell disease. The number of people annually diagnosed with the above exceeds 300 million, where the increased risk for bacterial co-infections^1,2^ can easily result in life-threatening conditions. Due to the rapidly rising number of registered cases of infections with antibiotic resistant bacteria, these people are highly susceptible to potentially lethal outcome. Upon hemolysis, free heme becomes available, allowing interactions with components of the local environment. It has recently been demonstrated how heme can paralyse macrophages^2^, key cellular elements of the immune system, however little is known about its interaction with other members, such as the vital humoral components of the innate immune system. In this respect, one important set of compounds are membrane active antimicrobial peptides (AMPs), also called host defense peptides (HDPs) produced by the host organism. Note that in this study we use both terms, where AMP refers to their direct antibacterial activity while HDP highlights functions beyond.

Considering the outlined importance above, in the present study we aimed to explore the interactions between free heme and AMPs. The latter are diverse peptides with the ability to attack pathogens like bacteria or fungi, acting thereby as an effective tool in the fight against invading microorganisms^3–5^. AMPs show structural diversity, and based on common characteristics, they can be divided into several classes^6^, among them the linear cationic amphipathic peptides^7^ represent a populated group with many examples from organisms ranging from insects, through amphibians to vertebrates^8^. They target the integrity of microbial membranes, though related diverse effects including immunomodulatory roles were also demonstrated^9–12^. Membrane disruption can be achieved in several ways^3,13^, where AMP binding is facilitated by electrostatic attraction to the negatively charged microbial membrane, and hydrophobic contacts contribute to membrane insertion^14,15^. Folding together with peptide accumulation upon membrane interaction is believed to be a key step in AMP action^16^. However, very little is known about potential non-lipidic partners that are capable of modulating peptide structure and function. In this regard, helical conversion and assembly formation of AMPs could be induced by several sets of small molecules including synthetic dyes, marketed drugs, or even endogenous metabolites^17–20^. Related studies show that complex formation with the small molecule partners can result in blocking^21^, attenuating^22^, or even enhancing^23^ peptide activity. This is contrasted to enhanced antimicrobial efficiency demonstrated with therapeutic antibiotics where peptides and small molecules act separately even when administered in combination^14,24^.

In this respect heme as an endogenous small molecule is also relevant. It is a key prosthetic group in various transport proteins^25^ and enzymes^26^ involved in numerous vital physiological and metabolic processes. Beyond its well-known functions, new roles e.g. in signalling have also been demonstrated^27^. Free heme, released from hemoglobin upon hemolytic events exerts proinflammatory effects^28^, and contributes to production of reactive oxygen species, lipid peroxidation, and intracellular oxidative stress^29–31^. Further on, both cytotoxic effects^32,33^ and induced cytoprotective^34^ responses of heme and its oxidized form, hemin, were reported, indicating that these compounds may have a balancing effect between these two directions^35^. The cytoprotective activity is linked to the inducible heme-degrading enzyme heme oxygenase-1 producing biliverdin, free iron, and carbon monoxide, where hemin can act as the inducer of the protein. In contrast to the parent heme, biliverdin is an effective antioxidant^34^. Structurally, heme is composed of a porphyrin ring coordinating a Fe^2+^ ion, and two propionyl side chains (Fig. S1). In biliverdin, the central iron is lacking, and the oxidized porphyrin ring is open (Fig. S1). It should be noted that hemin is a lipophilic molecule with the ability to disorganize lipid bilayers^33^ and membrane skeleton^31^.

To further understand the impact of AMP-small molecule interactions on structure and function, here we employed an extended set of membrane active peptides (Table 1) including human-derived sequences to explore their binding to hemin and biliverdin. Our previous studies have indicated the helix inducer effect of hemin on some selected AMPs^19,20^, however, these relations, especially in the presence of lipid bilayers, vital components for AMP function, have not been explored yet. Here we show that hemin can control structure and assembly of basic amphipathic peptides. Moreover, complex formation with hemin affects peptide membrane activity as demonstrated in binding assays with model membranes mimicking properties of mammalian and bacterial cell membranes. On the other hand, toxic effects of free heme could be attenuated via sequestering by HDPs.

**Table 1:** Sequence and properties of the membrane-active peptides used.

Peptide	Source	Sequence	Residue	Net charge
Melittin	Insect venom	GIGAVLKVLT TGLPALISWI KRKRQQ	26	+ 6
CM15	Synthetic hybrid	KWKLFKKIGA VLKVL	15	+ 6
LL-37	Human (skin, intestine, etc.)	LLGDFFRKSK EKIGKEFKRI VQRIKDFLRN LVPRTES	37	+ 7
FK-16	LL-37-derived, synthetic	FKRIVQRIKD FLRNLV	16	+ 5
Buforin I	Frog stomach, histone-derived, Human gastric fluid^a^	AGRGKQGGKV RAKAKTRSSR AGLQFPVGRV HRLLRKGNY ^a^	39	+ 13
Dhvar4	Human saliva histatin-derived synthetic	KRLFKKLLFS LRKY	14	+ 7
Macropin 1	Insect venom	GFGMALKLLK KVL	13	+ 4
Lasioglossin III	Insect venom	VNWKKVLGKI IKVAK	15	+ 3
Temporin-La	Frog skin	LLRHVVKILE KYL	13	+ 6

For helical wheel representation of the peptides see Fig. S1. Note that all peptides are amidated at their C-terminus. Net charge refers to pH 7.

^a^This is the bullfrog sequence^36^, the human variant is *S*GRGKQGGK*A* RAKAK*T(S)*RSSR AGLQFPVGRV HRLLRKGNY according to the uniprot website (https://www.uniprot.org).

## Results and discussion

### Peptide selection

To address interactions with heme compounds, we tested nine membrane-active peptides belonging to the class of linear cationic helical AMPs (Table 1). The primary focus is set on natural human sequences distributed throughout the body such as the only human cathelicidin LL-37, a HDP with various immunomodulatory effects^37^, also testing its active fragment FK-16^38^. AMPs along the gastro-intestinal tract were also considered, such as buforin I, a human gastric fluid AMP^39^, and Dhvar4, a potent AMP variant developed from human saliva histatins^40^. For the latter, interaction with food color small molecules affecting its membrane activity has been reported^23^. As reference, peptides already investigated for hemin interaction in the absence of lipid bilayers^19^ such as the bee venom component melittin and its hybrid CM15 were also studied. The former two peptides are interesting because of their strong and moderate hemolytic activity, respectively. As insect venom get into contact with the blood of the target organisms and activate their immune responses^41^, we selected further related venom-derived AMPs, macropin 1 and lasioglossin III. Moreover, we also involved temporin La, a member of the populated temporin family of frog skin-derived short AMPs, which show similarities in chemotactic and histamine-releasing properties to insect venom AMPs^42^.

### Interaction of hemin with membrane-active peptides: folding coupled to assembly

**Figure 1:**
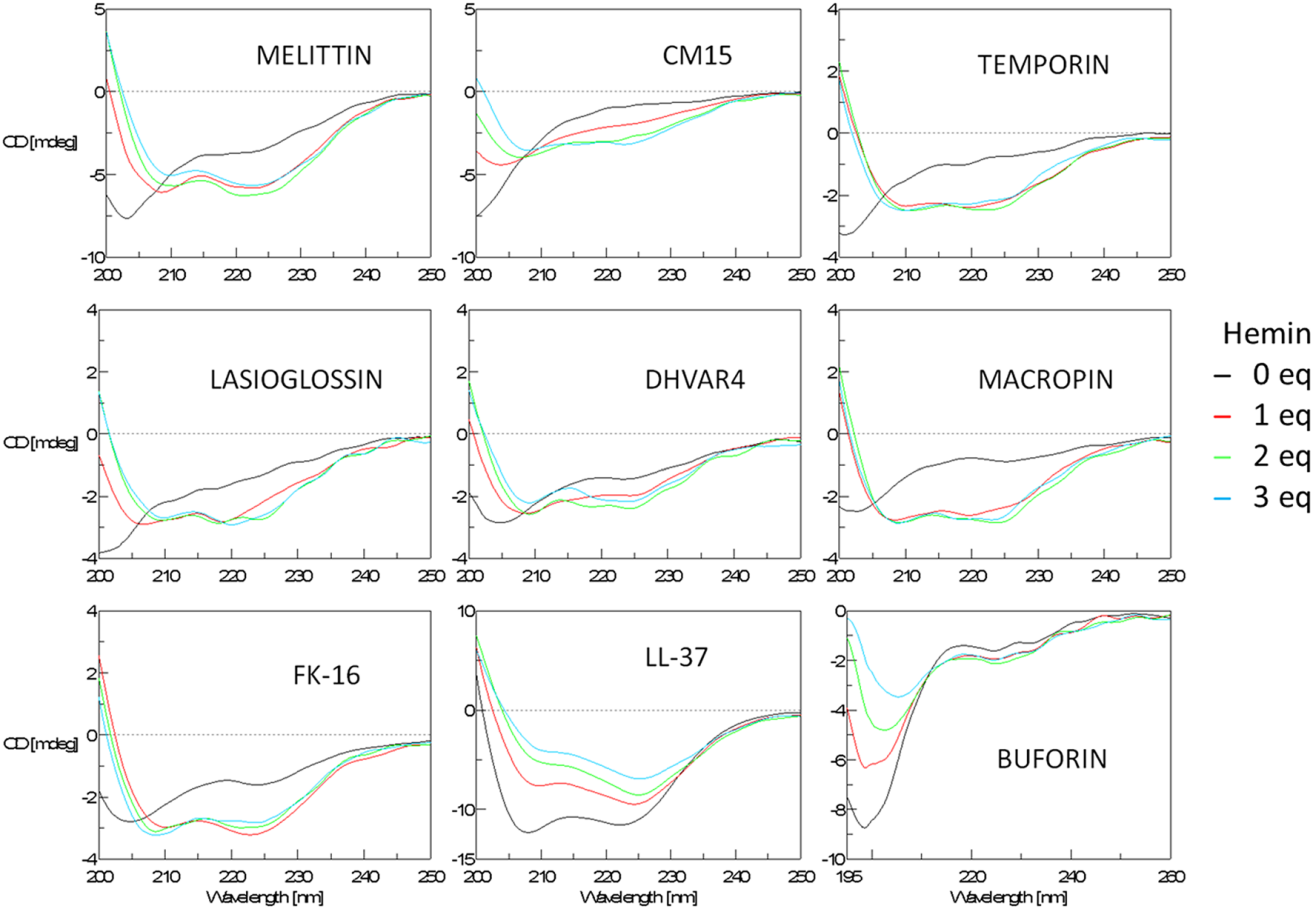
CD spectral changes of the peptides upon hemin titration. Spectra were collected at 25 µM peptide upon consecutive addition of molar equivalents (eq) of hemin in PBS. For better visual inspection non-uniform ellipticity scales are applied. The induced secondary structure is helical for most peptides except for buforin that retains significant disorder. The overall intensity is reduced for melittin, CM15, temporin, lasioglossin, and Dhvar4, compared to the higher relative signals for macropin, and FK-16. The helical character of LL-37 is preserved in the presence of hemin.

Potential conformational changes upon peptide interaction with hemin were addressed with CD spectroscopy. Without any additives, most peptides exhibited a CD spectrum with a pronounced negative peak at around 200 nm (Fig. 1) according to the disordered state. As an exception, LL-37 adopted an α-helical conformation (Fig. 1), which is in line with a folded state reported at higher ionic strength^20^, also employed here. Upon subsequent addition of hemin, the spectral changes are indicative of enhanced ordered conformation induced for all the peptides except buforin (Fig. 1). The resulting helix-rich state is evident from the characteristic spectral features, i.e. the double minima at about 208 and 222 nm. These extrema, however, are slightly red shifted (by 1–3 nm) indicating some β-sheet content and aggregation as suggested previously^19^. Based on these results, the helical conversion induced by hemin seems to be a general phenomenon for basic amphipathic membrane-active peptides. Considering buforin, its sequence cannot fit into a regular amphipathic helix (Fig. S1), which can at least partially explain the lack of helix formation.

**Figure 2:**
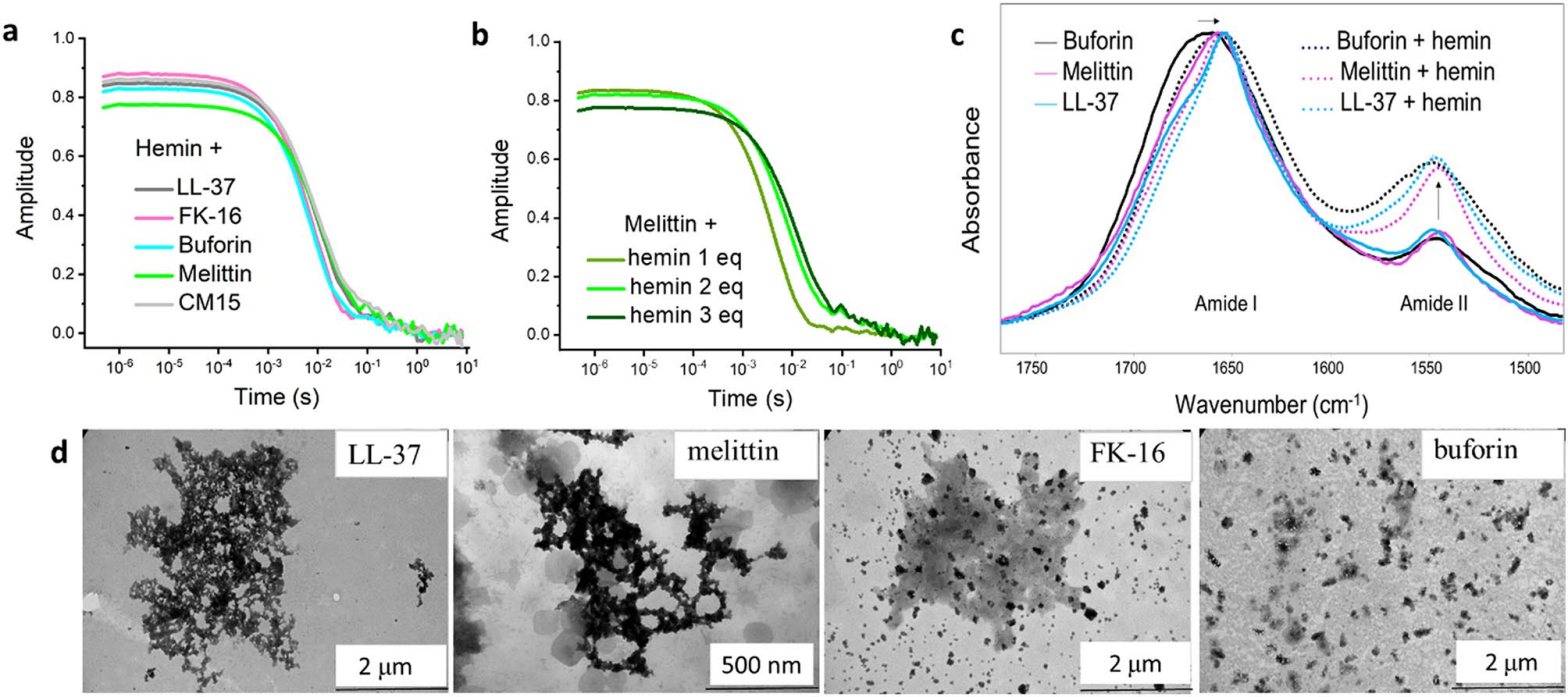
Assembly formation of peptide-hemin complexes. (**a**) DLS correlation functions detecting peptide-hemin co-assembly in the high nanometer—low micrometer scale. Peptide and hemin concentration was 25 μM and 75 μM, respectively. (**b**) Typical DLS correlation functions measured for peptide-hemin systems upon subsequent addition of hemin molar equivalents (eq) to the peptides, shown here for the titration of melittin (25 μM). (**c**) Amide I and II regions of the ATR FT-IR spectra collected for dry films formed from a solution of 25 µM peptide in the absence and presence of hemin at 1:3 peptide-to-hemin ratio. Arrows indicate shifts of the amide I band and elevated relative amide II band intensities in the complexes. (**d**) TEM images of peptide-hemin aggregates show particles in increasing size and complexity in the order buforin < FK-16 < melittin < LL-37. Scale bar is indicated.

Furthermore, variations in CD signal intensities for the hemin-complexed peptides were also observed. This might be linked to differences in the helix content, however, signal loss could also indicate different levels of co-assembly formation and/or adsorption of the hemin complex to the cuvette. Indeed, higher-sized particles were readily detected by dynamic light scattering (DLS) (Fig. 2a). Upon hemin addition to the helical LL-37, the overall signal decrease, relative gain in intensity of the 222 nm band, and concomitant red shift of the minima together are compatible with preserved helical structure, but the backbone is buried within a more hydrophobic environment of the peptide assembly. FK-16, the active fragment of LL-37, also showed the disordered-to-helix conversion, and the highest signal intensity is indicative of less/lower sized associates. Among the short peptides, macropin behaved similarly. In contrast, the long buforin was affected by hemin in a different way. The classical spectral features of the helical conversion were not detected, and the unfolded state remained dominant, nevertheless, the moderate red shift of the minimum and the signal loss indicated some gain in ordered structure content and formation of supramolecular co-assemblies as well.

As CD spectra could easily be perturbed due to the presence of the assemblies formed, we turned to a technique with signals non-sensitive to aggregation^43^. We exploited infrared spectroscopy with the ATR accessory where dry film samples can be investigated. Conditions in the dry film mimic a crowded environment and thereby report on the intrinsic helix-forming ability of locally accumulated peptides as well. Melittin, CM15, LL-37, FK-16 and buforin showed peculiar conformational behaviour in CD measurements (Fig. 1), among which we selected the long ones, melittin, LL-37, and buforin, displaying spectra with good signal to noise ratios. Based on amide I band maximum position and band width (Fig. 2c)^44^, buforin preserved a dominant unfolded conformation in the concentrated dry film while increased helix content was detected for melittin known to form helical oligomers at higher concentrations^45^, and LL-37 showed the highest helicity with the narrowest band centred at ~ 1655 cm^−1^ (Fig. 2c). Upon hemin addition at a peptide-to-hemin ratio of 1:3, the more folded state in the complex was evident for all three peptides from the band sharpening, and/or spectral shifts towards 1655 cm^−1^ (Fig. 2c), the characteristic position for the helical conformation. The most noticeable change covered by a shift from 1664 to 1656 cm^−1^ was detected for buforin, suggesting a more structured conformation in the hemin-complex, observed not so markedly with CD spectroscopy. For the helical LL37, intensity loss of the high wavenumber shoulder (Fig. 2c) might be indicative of some arrangement of the helices in the hemin-complexed state.

The elevated relative amide II signal resembling spectral features of larger proteins reports on formation of assemblies with protein-like structures^46^ in the hemin complex for all three peptides (Fig. 2c). This is in line with DLS results indicating particles typically in the 1–2 µm scale for each peptide of the set used (Fig. 2a). The aggregate size was detected already upon addition of one molar equivalent of hemin (Fig. 2b). However, differences in size and morphology of the peptide-hemin assemblies were also revealed using TEM, which could be correlated with conformational changes. In the TEM images of the hemin complexes (Fig. 2d), the most helical LL-37 exhibited the most well-defined overall structure with the highest complexity, followed by melittin with comparable properties, whereas the softest, loosest aggregate state was detected for the less ordered buforin. The short FK-16 showed an intermediate behaviour forming particles with both features. Similar morphology has already been observed for peptide associates formed with various binding partners such as LL-37 with RNA^47^, CM15 with the therapeutic drug suramin^21^, or Dhvar4 with the food colour tartrazine^23^. All these findings suggest that formation of such semi-folded assemblies might be common in interactions of amphipathic peptides with natural or synthetic small molecules. Related recent structural insight to self-assembled states of LL-37 and melittin also indicate that these peptides have the capacity to form supramolecular scaffolds with high internal order^48,49^.

### Effect of hemin on peptide membrane activity

To test whether hemin is able to access peptides associated to target membranes, we addressed the effect of hemin in the presence of model vesicles. Phosphatidylglycerol (PG)-containing liposomes were used to mimic the negatively charged surface of natural bacterial membranes. As AMPs can harm host cells, effects were also measured with pure phosphatidylcholine (PC) liposomes built up of zwitterionic lipids mimicking the neutral outer leaflet of mammalian cell membranes.

Utilizing CD spectroscopy, hemin titration of lipid-bound peptides was carried out with five peptides (melittin, CM15, LL-37, FK-16, and buforin, Fig. S2) selected based on their conformational behaviour in the absence of lipids (see above). It is well-known that melittin shows high affinity to both neutral and negatively charged lipids as reflected in its pronounced hemolytic activity^50^. In line with this, it adopted helical conformation in the presence of both PC and PC/PG liposomes, although the helix content was somewhat higher with PC/PG (Fig. S2a). Upon addition of hemin to the helical lipid-bound peptide, the structural change was still clearly detectable as the minima shifted to lower wavelengths, characteristic for the hemin-bound state. The synthetic hybrid variant of melittin, CM15 exhibits reduced hemolytic activity^51^ as it has increased selectivity to negatively charged lipids. According to this fact and due to its shorter chain, only partial helix formation is observed upon lipid binding, particularly with PC, indicated by weak signals at 222 nm (Fig. S2c). However, helicity is further enhanced upon hemin addition resulting in a conformation resembling the hemin-bound state again. Lipid interaction of the helical LL-37 lead to elevated helicity in the presence of PC/PG only (Fig. S2d), however, hemin was able to affect peptide conformation in a similar way as in the absence of lipid bilayers. For its fragment FK-16, binding to PC increased the helix content, and hemin addition resulted in spectra similar in shape to the no lipid case but with higher signal intensity (Fig. S2e). In contrast, no spectral shift but intensity variation only was detected for the PC/PG-bound peptide suggesting that hemin cannot access the peptide when strongly bound to the lipid bilayer. As in the absence of lipids, buforin behaved exceptionally also in the presence of model membranes (Fig. S2b). None of the liposomes was able to induce detectable ordered conformation suggesting no lipid interaction, thus hemin titration resulted in CD curves similar to those measured in the absence of lipids. The overall signal intensity loss, characteristic for extensive aggregation upon association to hemin, was also clearly observed for melittin, CM15, and LL-37 even in the presence of liposomes. Results on the selected peptides point out that interaction with hemin could overrule membrane binding. It is to be noted that the least significant intensity loss was observed with the lipid-bound FK-16, for which the highest CD signal was detected upon hemin binding in the absence of liposomes.

**Figure 3:**
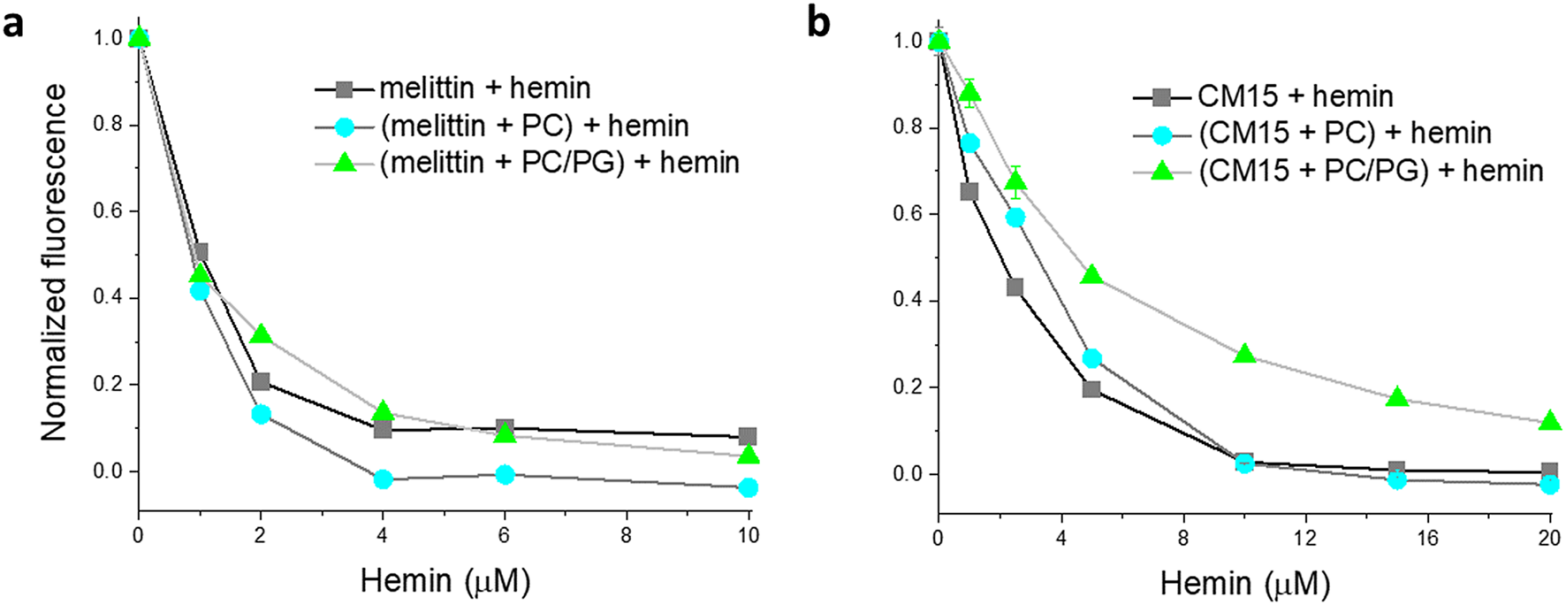
Fluorescence titration of selected peptides with hemin in the presence and absence of model membranes. Titrations were performed with peptides containing a Trp residue, melittin (**a**) and CM15 (**b**). Spectra were collected at 2 µM peptide with or without 635 µM lipid, using consecutive addition of hemin in PBS. Data are read at the emission maximum, corrected for corresponding values of the blank titration, and normalized to values measured in the absence of hemin.

Effect of hemin on the lipid-bound peptide was also addressed following intrinsic tryptophan fluorescence. Using melittin, we have demonstrated previously that hemin and several heme derivatives could reduce peptide fluorescence via quenching the signal^19^. Among the compounds tested, the iron-containing hemin was the most effective quencher with a remaining intensity of ~ 5–10% at a hemin-to-peptide ratio of 1:3^19^. These features were also reproduced here, furthermore, the administration of hemin to lipid-loaded melittin resulted in similar titration curves (Fig. 3a). In the presence of PC liposomes, quenching was even more effective leading to complete signal loss whereas the binding strength remained apparently the same as was without lipids. This might be attributed to simultaneous binding of hemin to the lipid bilayer. For the PC/PG-bound peptide, hemin affinity slightly reduced (Fig. 3a), which can be explained by the electrostatic repulsion of hemin from the negatively charged lipid and/or tighter peptide binding to PG. These data indicate that hemin can access membrane-associated peptides, likely resulting in complexes incorporating all three components, namely hemin, the AMP and the membrane. For CM15, quenching led to total signal loss at 10 µM hemin (Fig. 3b), presumably due to a closer contact between the Trp of the short peptide and the porphyrin ring in the complex. Very a similar titration curve was obtained in the presence of PC liposomes indicating the low affinity of the peptide to neutral vesicles again. In contrast, hemin caused significantly lower quenching on the PC/PG-bound peptide, in agreement with stronger lipid-peptide interaction. Additionally, the lower affinity of hemin to charged lipids could also contribute to the reduced effect.

**Figure 4:**
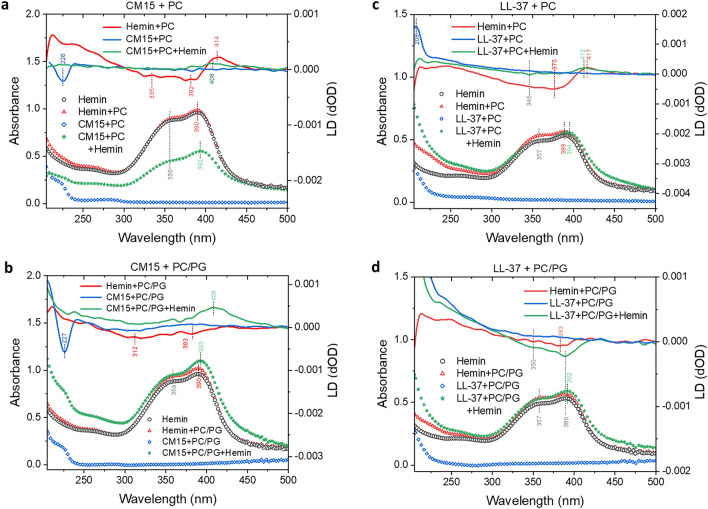
Oriented binding of hemin to model membranes in the presence and absence of peptides. LD and absorbance spectra were collected in PBS supplemented with 50% sucrose at peptide, hemin and lipid concentration of 85 µM, 255 µM and 1.3 mM with CM15 (**a,b**) or 50 µM, 150 µM, and 1.3 mM with LL-37 (**c,d**), respectively. In the three-component system, hemin was added to liposomes preincubated with peptides.

Based on the above examples of melittin and CM15, response of the peptides to hemin can vary. This was further supported by results obtained from flow-LD experiments. Linear dichroism is a technique suitable to provide information of the oriented binding of chromophores to lipid vesicles aligned by applying external shear force^52^. The ability of hemin to bind to neutral lipid bilayers in an ordered fashion was indicated by the emergence of a broad negative peak in the 300–400 nm region and a sharper positive peak centred at ~ 415 nm (Fig. 4a,c), corresponding to the Soret-band region of the heme chromophore. Based on assignment on membrane-associated heme-containing proteins^53^, this might reflect a binding geometry where one of the two orthogonally polarized transition moments lying in the plane of the porphyrin ring points rather towards the membrane normal whereas the other one is aligned parallel to the membrane surface. Oriented hemin was observed even when hemin was introduced to PC-bound LL-37, however, the shape of the Soret-band changed significantly so that only a single positive peak at ~ 410 nm was detected (Fig. 4c). As this peak is characteristic for heme in heme-binding proteins, this could be attributed to membrane associated peptide-hemin complexes. In contrast, the lack of LD signal for the CM15-hemin-PC system (Fig. 4a) indicated the loss of oriented hemin binding. The LD spectrum of hemin with PC/PG liposomes displays a low intensity broad negative peak suggesting a loosely associated, more randomly oriented hemin (Fig. 4b,d). Interestingly, more pronounced peaks were observed for the peptide-hemin-lipid systems, characterized by a single positive and a single negative peak at ~ 410 and at 350–400 nm with CM15 and LL-37, respectively. It seems that in the subpopulation of membrane-bound CM15-hemin assemblies, hemin is positioned rather parallel to the membrane surface whereas in the LL-37-hemin co-assembly, the preferred orientation for hemin is perpendicular to that. The rather sharp absorbance peak at 390 nm over that at 350 nm within the envelope of the Soret band (Fig. 4a–d) suggests that hemin is present as monomers and dimers/higher associates^54–56^, and the lack of remarkable spectral changes indicate that the interaction with lipid bilayers does not shift this equilibrium significantly. Upon binding to vesicles covered by peptides, the monomeric hemin population is preserved, or even slightly increased with PC/PG.

The far UV part of the LD spectra (Fig. 4) corresponds to peptide amide bonds of membrane-bound helices^52^. Stronger binding of a more helical peptide to PC/PG over PC is clear based on higher LD signals in this range. In the three-component systems, liposome-associated helices were not detected with PC, however, a fraction of the peptides retained their PC/PG-bound orientation even upon addition of hemin. As the Trp side chains also contribute to this region, the Trp residue in CM15 could also be monitored based on the characteristic LD peak of the *B*_*b*_ transition of the indole chromophore at 225 nm. The intense negative signal is indicative of membrane-inserted Trp side chain, which disappeared in the presence of hemin with both PC and PC/PG (Fig. 4a,b). Combined LD data suggest that hemin-complexed peptides could retain their lipid-bound state, however, hemin is able to change the overall orientation of the peptides including lifting out the anchoring Trp side chain.

### Relative affinity of the peptide interactions in peptide-hemin-lipid systems

Based on CD spectral features, apparent dissociation constants in the 20–50 μM range could be estimated for the peptide-hemin interactions as a single dose of a molar equivalent of hemin (25 μM) was enough here to reach close-to-saturation levels for several peptides (Fig. 1). Somewhat lower binding strength of 60–120 μM has been determined for the interaction of melittin with hemin^19^ while the K_d_ value of 20–36 μM calculated for the LL-37–hemin interaction^20^ agrees well with the above estimates. It should be noted that classical considerations on affinity determination might fail for the present heterogeneous complexes with a fairly non-evident stoichiometry. Therefore, here we address relative affinities rather qualitatively in the three-component systems composed of peptides, hemin and model membranes, monitoring peptide partition upon varying the mixing order of the components.

Structural changes were analysed for CM15 where the lipid- and hemin-bound features are easy to distinguish. For all three-component systems, spectra characteristic for the hemin-complexed peptide were observed independent of the mixing order (Fig. S5a,b), which suggests the prevalence of peptide-hemin interaction even in the presence of membranes. Nevertheless, intensity variations allowed us to categorize levels of hemin-induced associate formation as follows: (lipid + peptide) + hemin < (lipid + hemin) + peptide < (peptide + hemin) + lipid < peptide + hemin. This order applies to both PC and PC/PG liposomes. Aggregated particles dominating over the scattering of the vesicles were observed with DLS (Fig. S5c,d). Interestingly, and somewhat unexpectedly, less and/or lower-sized particles were detected in mixtures where peptides met first the small molecule followed by the lipids. More peptides remained lipid-bound in the presence of anionic PC/PG while pronounced assembly formation was found with the neutral PC liposome upon subsequent addition of hemin. The latter mixtures resembled most the hemin-loaded state without membranes. These data point to a dynamic system where hemin could bind to the lipid-associated peptide resulting in an assembly including all three binding partners, nevertheless, the peptide-small molecule complex might also associate to the lipid bilayer.

**Figure 5:**
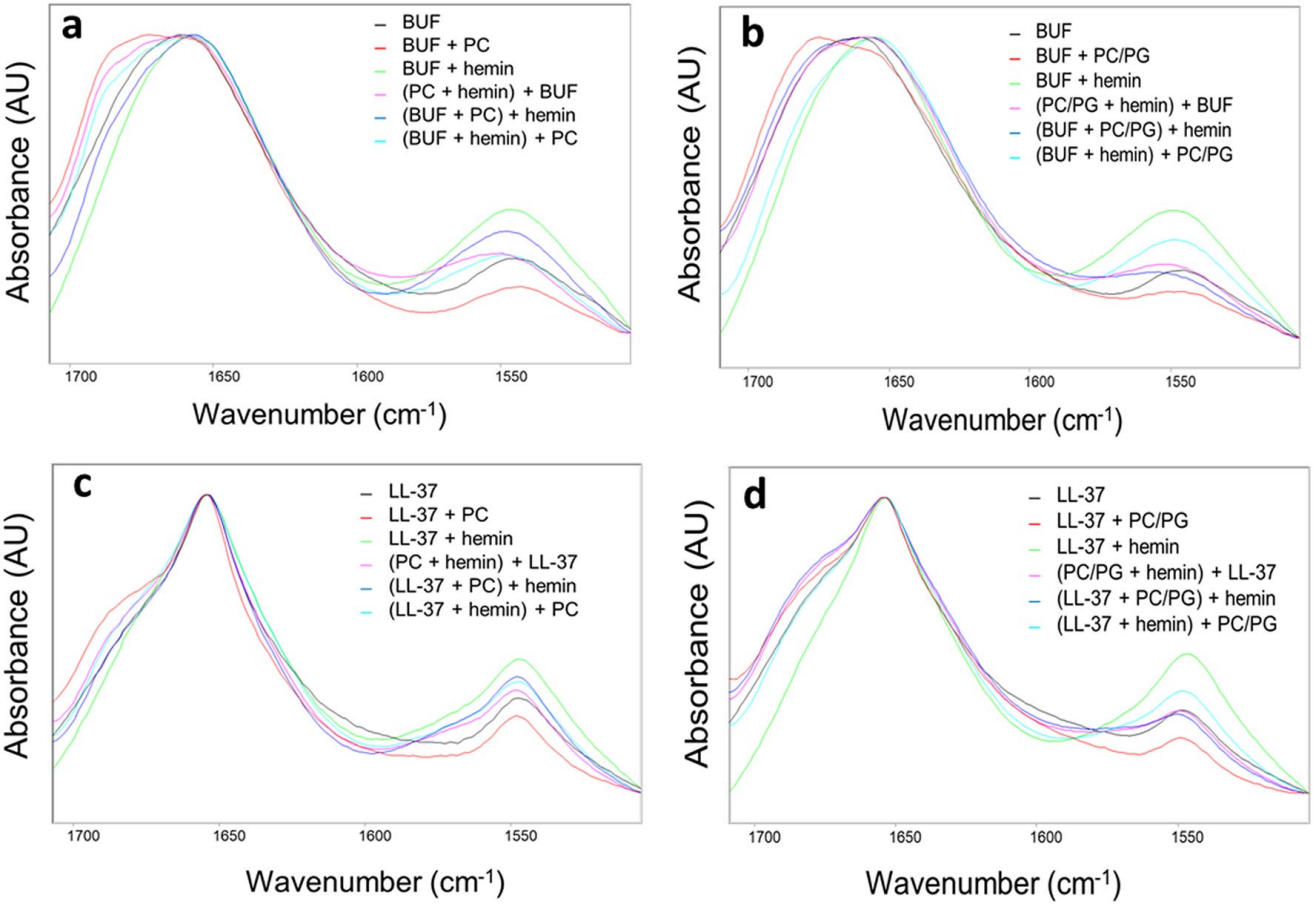
IR analysis of peptide partition in the three-component systems based on peptide amide bond vibrations. Amide I and II regions of the spectra are shown collected for dry films obtained from 25 µM peptide, 75 µM hemin, and 635 µM lipid. Spectra are normalized to amide I intensities. *BUF* buforin.

IR analysis on the three-component systems revealed further binding preferences for buforin and LL-37. Peptide partition in the three-component mixtures was evaluated based on two spectral features (Fig. 5, Table S2), i.e. the intensity of the emerging amide I component at ~ 1680 cm^-1^, characteristic for the lipid-bound state (Fig. S6), and the increased relative amide II band intensity, indicative of peptides aggregated to protein-like structures in their hemin-complexes. As a general trend, the highest fraction of the protein-like assembly was observed when the preformed peptide-hemin complex was introduced to the liposomes (Fig. 5b–d, cyan lines). In these mixtures, the membrane-bound peptide fraction still could be detected to various extent, least for buforin with PC/PG (Fig. 5b). In the latter case, the preserved hemin-associated state precluded formation of the membrane-active conformation. Moreover, buforin represented an exception to the above trend as well, as unexpectedly high amount of the highly aggregated form was found only when hemin was added to the peptide preincubated first with PC (Fig. 5a). In the buforin-PC system, buforin preferred the membrane best upon addition to the PC-hemin mixture where hemin was presumably associated to the lipid bilayer. Although buforin could adopt its lipid-bound conformation (Fig. 5a, amide I region), some contact to the PC-associated hemin is also indicated by the shifted amide II band maximum. Such a shift was also detected for buforin with PC/PG (Fig. 5b). For LL-37, the membrane-bound fraction was more dominant with PC/PG than with PC (Fig. 5c vs d), which is in line with preferred peptide binding to PC/PG. However, the common membrane-bound, presumably active conformation is hardly detected when the LL-37-hemin complex is introduced to PC/PG, and a free peptide-like conformation is observed instead even when the protein-like assembled state is partially preserved (Fig. 5d).

In summary, while peptide partition between lipid-bound and hemin-complexed states was found specific for each peptide studied, some general trends could also be revealed. Particularly, the charge of the lipid bilayer played a pivotal role correlating with peptide lipid selectivity and helix formation ability. Further on, the mixing order of the components affected peptide partition significantly, demonstrating that preformed peptide-hemin complexes do not entirely dissociate even in presence of a lipid membrane that is normally favoured by that particular AMP.

The interaction network studied here was further characterized analysing lipid vibrations corresponding to the acyl chain, the ester neck, and head-group phosphate or choline (Table S1, S3–S5, Figs. S7–S11, and related text). Results are in concert with the above data and contribute to better understanding the effects of membrane-active peptides and small molecule agents exerted on lipid bilayers.

### Effect of biliverdin in comparison with hemin

As hemin induces production of biliverdin, and biliverdin exerts opposite inflammatory effects compared to hemin, we also probed interactions with biliverdin. For the extended set of peptides, biliverdin triggered conformational changes (Fig. S3) in a similar way as observed for hemin (Fig. 1). However, biliverdin induced no significant helix formation in some peptides, particularly the shorter ones, such as temporin or macropin, for which gain in ordered conformation was clear with hemin. Comparing the affinity, higher dosage of biliverdin over hemin was needed to reach saturation. In previous works^19^, higher binding strength has been determined for biliverdin over hemin with melittin. The difference might be attributed to higher levels of hemin oligomers likely present in PBS used here to mimic physiological conditions, which could promote peptide binding and assembly.

**Figure 6:**
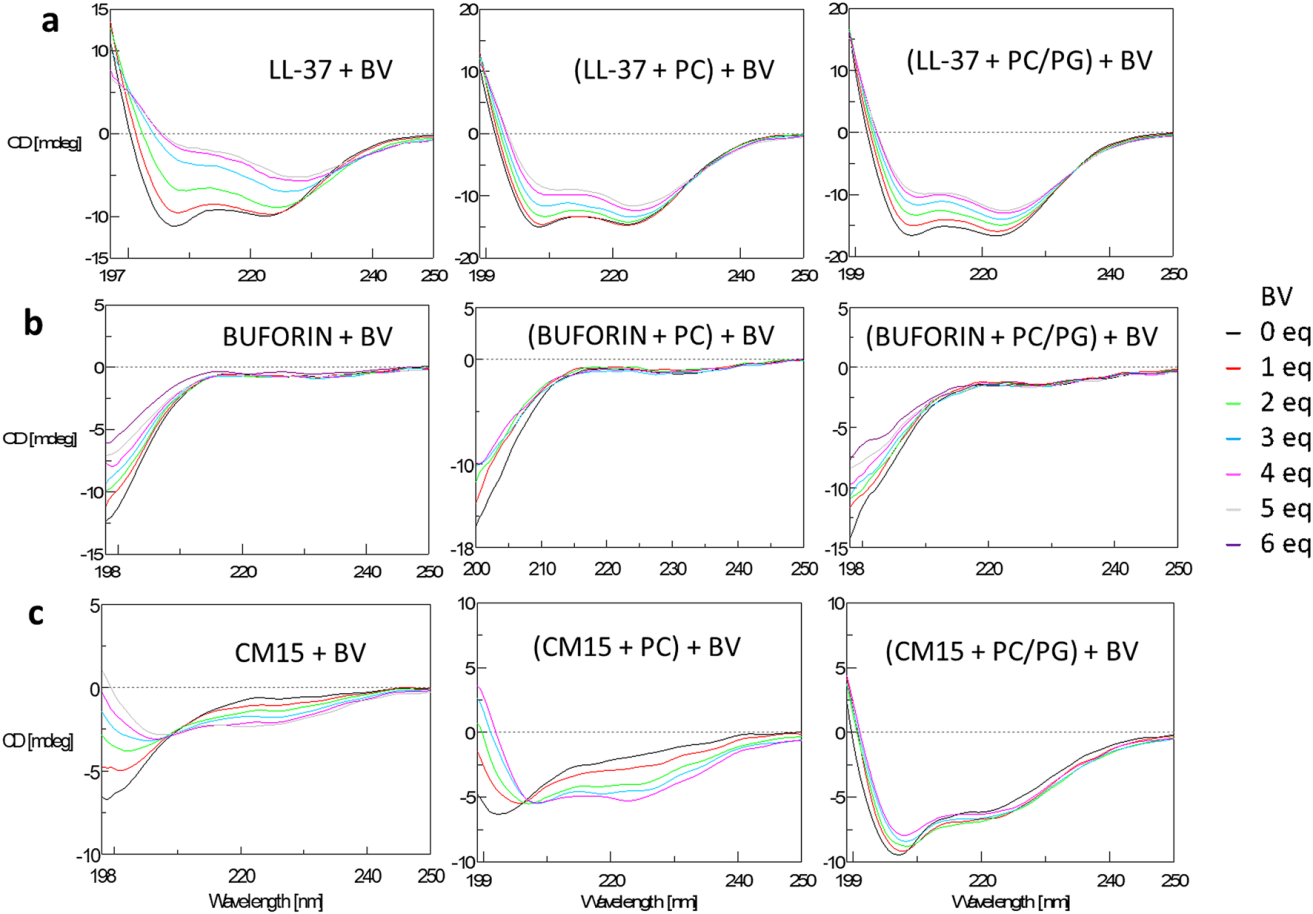
CD spectral changes of CM15 upon titration with biliverdin in the presence and absence of model membranes. Spectra were collected at 25 µM peptide and 635 µM lipid upon consecutive addition of molar equivalents (eq) of biliverdin (BV) in PBS.

The effect of the biliverdin on peptide membrane activity was examined on selected peptides using CD titrations. Similar overall characteristics was observed for the biliverdin-peptide-lipid systems (Fig. 6, Supplementary Fig. S4) as obtained for the hemin systems, however, the concentration required for the same effect was higher. A further difference was revealed with CM15. This short AMP is highly specific to negatively charged membrane triggering definite helix formation. Although hemin was able to access the lipid-bound peptide as indicated by highly reduced CD signals upon addition of hemin to PC/PG-bound peptides (Fig. S2c), biliverdin hardly affected signal intensity (Fig. 6c) suggesting no significant perturbation of membrane-associated CM15 by biliverdin.

### Impact of hemin and biliverdin on the cytostatic effect of the peptides

Our in vitro binding assays indicated that interactions with hemin can effectively promote peptide helical conformation, formation of larger assemblies, and alter peptide binding to model membranes. To explore the biological relevance of these interactions on HDP action, viability assays were performed addressing their cytotoxic and cytostatic effects. Engineered hemin-sequestering peptides of bacterial origin were recently reported to affect heme availability on non-small cell lung cancer cells^57^, so the human lung squamous cell carcinoma derived EBC-1 cell line was selected for our tests. Our focus here was on the human cathelicidin peptide, LL-37 that is also expressed in mucosal epithelium in the lung^58^.

**Figure 7:**
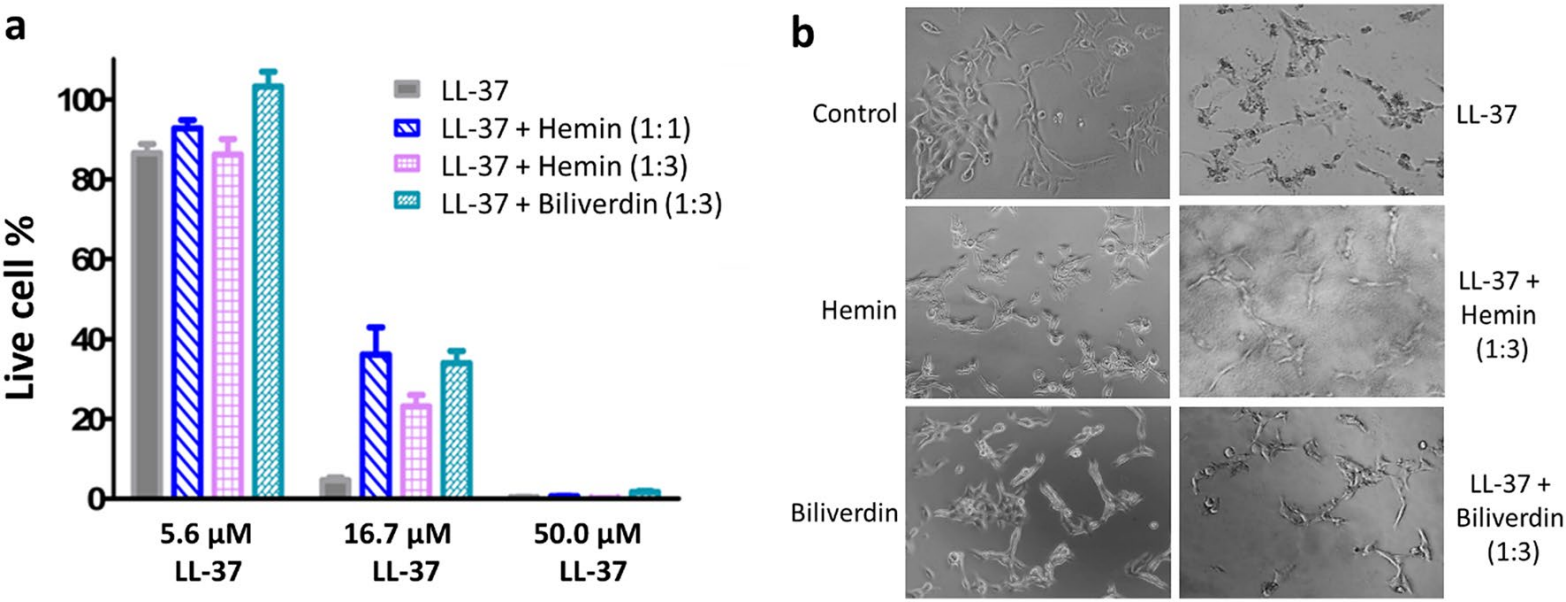
Cytostatic and cytotoxic effects on EBC-1 cells. Viability assays were performed on EBC-1 cells in the presence and absence of LL-37 and hemin or biliverdin as described in the “Materials and methods” section. (**a**) Live cell percentage after treatment with LL-37 at the indicated peptide concentrations alone or mixed with hemin or biliverdin at peptide to small molecule molar ratios of 1:3, or with hemin also at 1:1 ratio. Live cell percentage was calculated in relation to untreated control cells. Data are mean ± SEM, n = 3. (**b**) Bright field microscopic images of EBC-1 cells after 3 h treatment with LL-37 (17 µM), hemin or biliverdin (33 µM), or LL-37 (17 µM) premixed with hemin or biliverdin. Note the dark spots observed in the background around the cells for LL-37 + hemin (1:3), indicating formation of peptide-hemin associates.

LL-37 displayed moderate cytostatic effect on EBC-1 cells with no significant effect at ~ 5 µM while killing ~ 90% and almost all cells at 17 µM and 50 µM, respectively (Fig. 7a). No cytostatic effect was detected for biliverdin up to 100 µM whereas hemin alone was found cytostatic to EBC-1 cells at concentrations > 10 µM, with ~ 20% and 60% dead cells detected at 33 µM and 100 µM, respectively (Fig. S12). This is in line with toxic effects of hemin in this concentration range reported on various cells^59,60^. The cytostatic effect was significantly reduced when 17 µM peptide was administered together with hemin or biliverdin as live cell percentage increased from ~ 5 to ~ 25–35% (Fig. 7a). Hemin was effective in reducing the cytostatic effects even at 1:1 peptide to hemin ratio (36 ± 12% live cell %), moreover, hemin and biliverdin showed comparable effects at 1:3 ratio (live cell % was 23 ± 6% with hemin and 34 ± 6% with biliverdin vs 4.7 ± 1.3% for LL-37 alone).

Microscopic visualization of cell morphology changes upon treatment with LL-37 mixtures (Fig. 7b) supported the viability data above. Non-damaged cells were observed after 3 h exposure to hemin or biliverdin at 33 µM while the toxic effect of LL-37 at 17 µM was evident based on the noticed alterations involving shrinkage, and membrane defects. When treated with mixtures of peptide and hemin/biliverdin, the higher number of intact cells was obvious. Moreover, peptide-small molecule assemblies, particularly higher sized peptide-hemin supramolecular scaffolds were clearly seen around the cells, consistent with particles detected by TEM and DLS (Fig. 2).

These results are indicative of attenuation of peptide function by heme compounds. In the view of hemin, treatment with peptide-hemin mixtures resulted in cytotoxicity levels being between the values measured for the sole peptide and sole hemin solutions. This might indicate that not all peptides and hemin were complexed, and the action of the remaining free compounds was detected, however, it is also possible that peptide-hemin complexes might also target the cells. Considering the potentially negatively charged surface of cancer cells due to the presence of exposed phosphatidylserine (PS)^61^, the latter idea is not supported by our biophysical results above. Nevertheless, cancer cell lines do not evidently expose high levels of PS, indeed, only ~ 5 percent of ECB-1 cells was found positive in annexin V assays detecting specifically PS^62^. In this case, the mainly neutral lipid bilayer would allow binding of the peptide-hemin complex in line with our binding experiments with PC liposomes.

Based on our results on model membranes, and in vitro assays on the selected cell line above, further activity alterations of the AMPs upon complex formation with hemin may be speculated. First of all, AMP–heme interaction could result in reduced antimicrobial activity by lowering the active peptide concentration. This can be achieved either by sequestering the free available peptide fraction by heme not allowing membrane interaction or by precluding formation of the active membrane-bound conformation of the peptides in the hemin-complexed membrane-associated form. A further interesting point to be considered is possible relations between hemolytic activity and complex formation with hemin as AMP-induced hemolysis can also contribute to heme release. AMPs can be hemolytic to various extent where particularly melittin is known for its strong effects^63^, followed by CM15 designed to reduce the strong effect of melittin, still showing moderate hemolytic effects^63^, while other studied AMPs exhibit low toxicity on erythrocytes^63–69^. For the hemolytic peptides, the capacity to form complexes with the hemin released upon their action might be self-inhibitory thereby saving red blood cells from further lysis, also from the toxic effects of free heme.

### Characteristics of AMP-heme supramolecular interactions in comparison with heme-binding peptides

In common helical heme-binding proteins, heme is preferentially located in a hydrophobic pocket, where the ring is stacked to aromatic peptide residues, the central iron is coordinated axially to cysteine, histidine or tyrosine, rarely to Lys residues, however, the propionate moieties reside near to Arg or Lys peptide side chains^70^. Engineered peptides binding heme selectively, mimic this optimal binding site so that one or even more porphyrin rings are coordinated preferentially by His side chains arranged in ideal spatial distance provided by either helical^71–73^, β-sheet^74,75^, or β-hairpin^76^ constrained scaffolds. Considering the cationic-hydrophobic nature of the AMPs used here, although decorated with some aromatic groups but lacking Cys and mainly lacking His and Tyr as well, their sequential setup could provide some beneficial contacts for heme, however, they lack a well-defined heme binding site. Even in Dhvar4, the peptide derived from a His-rich histatin precursor, His residues are replaced during design to increase amphipathicity^40^. Moreover, even if buforin bears a His (His31 out of the 39 aa peptide), its exceptional behaviour could be attributed rather to its low helicity detected here. In summary, instead of a well-folded peptidic scaffold accommodating a (few) heme(s) at defined binding sites, our results suggest AMP-heme co-assemblies where heme is attached to the peptides via various energetically favoured contacts, but these contacts are not necessarily the same for each peptide within the associates. In this regard, AMP-heme co-assemblies may share features with peptide associates formed with various other small molecules. Indeed, recent in-silico approach has demonstrated for CM15 complexed with the polysulfonated, polyaromatic drug suramin that a combination of electrostatic, H-bonding, and cation-π interactions, together with aromatic stacking dictate positioning of the moieties in the complex^77^. Moreover, compounds bearing planar (aromatic) rings are prone to self-assemble, where an oligomer can serve as a platform to which peptides can accumulate. This way a couple of small molecules might mimic lower-sized macromolecules such as RNA, shorter DNA strands, or heparin, which are known to sequester AMPs easily^19,47,78^. The TEM images of these assemblies showing smaller clusters connected to larger associates support this idea. Alternatively, smaller peptide-compound assemblies might first form, which could further associate to higher aggregates. Indeed, the latter mechanism was revealed for the CM15-suramin system in recent computational studies^79^. These considerations are in line with results on designed heme-binding peptides where only highly optimized sequences formed a well-defined structure coordinating heme in a constrained binding geometry while less optimized peptides rather aggregated in the presence of heme^72^.

Current results are also relevant to nanotechnological applications as a potent, catalytically active transmembrane heme-protein could be developed based on a cationic helical AMP sequence^73^ related to those used here. Rational design of supramolecular co-assemblies has also an extreme potential in opening entirely new areas for biotechnological applications, however, these are in their infancy, where the above interactions could aid initial progress. Further on, heme-coordinating β-cage miniproteins made up of AMP-sized peptides exhibited K_d_ values below the nanomolar range, an extremely high affinity comparable to that of natural heme proteins^75^. Here we could estimate a binding strength typically in the low micromolar range for the hemin interaction with selected natural AMPs. Likewise, an apparent dissociation constant of ~ 3 μM was determined^73^ for the helical transmembrane scaffold designed based on a cationic AMP. Considering that free heme can reach higher micromolar levels under hemolytic conditions^80^, the micromolar affinity could be high enough for the interactions described in this work to happen in vivo. Nevertheless, AMPs cannot likely compete for heme with high affinity proteins.

### Conclusions and outlook

The function of linear cationic amphipathic AMPs is commonly linked to their conformational change into a definite helical active state. Herein we show on an extended set of AMPs that hemin is a potent helix inducer for this class of peptides, and complex formation with hemin results in assemblies with protein-like structures. We also illustrate that the complexity of the peptide-hemin associates correlates with peptide length and helicity. Moreover, the morphology of the peptide-hemin associates is shared with other peptide complexes formed with polyanionic partners with an aromatic scaffold pointing to a general mechanism of assembly formation driven by forces beyond mere electrostatic interactions. Several peptides could effectively associate with biliverdin as well, showing the characteristic features including helical conversion coupled to aggregation. However, the effect of biliverdin on shorter peptides was found less remarkable compared to hemin. This suggests that changes in the porphyrin ring flexibility, and the self-association propensity could have a key role in controlling their interactions with AMPs.

To judge the impact of heme on AMP function, membrane activity of the peptides was also considered. In this regard, the lipophilic nature also allows hemin to bind to various lipid membranes. Here we demonstrate in in vitro binding assays that assembly formation with hemin and biliverdin interferes with peptide membrane activity, and the effect is mainly dependent on peptide lipid selectivity and inherent helicity but also on their ability to self-associate or to form mixed aggregates. Importantly, peptide-hemin interactions could prevail even in the presence of lipid bilayers of various composition.

We have already reported examples where AMP interaction with various anionic amphiphilic small molecules could modulate peptide structure and activity. In line with these, our results obtained from in vitro cell-based assays indicated regulatory roles of heme-peptide interactions on cell viability. The toxic effect of LL-37 on EBC-1 cells was attenuated by hemin and biliverdin to a similar extent in concert with their comparable conformational impact on this peptide.

In summary, our in vitro findings suggest that direct interaction of HDPs with heme could control their in vivo action. These interactions can have potential relevance under conditions with elevated HDP and free heme levels, as follows.

- The immune response and related inflammation can be affected in several, partly opposite, ways, as both free heme and HDPs can exert diverse effects including recruiting immune cells vs inhibiting immune cell function^2,12,29^, and mediating pro- or anti-inflammatory responses^34,81,82^. The local free heme to HDP ratio at infection sites could contribute to determining the local defense response.
- Free heme can serve as iron source for bacteria^83^. Here we show that small, only 15–40 aa long peptides, with no dedicated binding site for heme, are also capable of capturing hemin, which could contribute to depleting locally available heme iron. Related to this, we have demonstrated the ability of LL-37 to target siderophores^17^, which are chelators secreted by bacteria to scavenge iron from the host environment. Thus, the same class of cationic peptides could block both direct and indirect iron acquiring strategies of bacteria.
- For prokaryotic and eukaryotic cells, the heme group is essential in many metabolic processes, which thus rely on heme availability. However, heme is known for its cytotoxic effects, likewise, overexpressed HDPs can also target host cells in a damaging manner. Through complex formation, HDPs could contribute to the clearance of local harmful heme, helping dedicated heme-binding proteins, such as hemopexin, which are commonly recruited to deplete excess heme^84^. On the other hand, binding to heme could attenuate unwanted peptide toxicity.
- Free heme and their metabolites biliverdin and bilirubin play diverse roles in oxidative processes^82^. As hemin and biliverdin was found to affect several AMPs to a comparable extent while others were sensitive rather to hemin, AMPs might shift the balance defined by the action of the heme compounds in oxidative stress conditions.

## Materials and methods

### Peptide, hemin, and lipid solutions

Melittin, LL-37, FK-16, buforin I, macropin 1, lasioglossin III, and temporin La (> 95% purity) were purchased from NovoPro (Shanghai, China). Peptides CM15, and Dhvar4 were synthesized in-house, and purity was > 95% according to LC–MS analysis. Peptide solutions were prepared in ultrapure water at 1 mM, aliquoted, and stored frozen at − 18 °C.

Hemin, and biliverdin HCl were purchased from Frontier Scientific. 1–3 mM stock solutions were prepared in water by titration with NaOH until complete dissolution.

High purity synthetic 1,2-dioleoyl-*sn*-glycero-3-phosphocholine (DOPC) and 1,2-dioleoyl-*sn*-glycero-3-[phospho-*rac*-(1-glycerol)], sodium salt (DOPG) was purchased from Avanti Polar Lipids Inc. (Alabastar, Alabama). Liposomes were prepared using the lipid film hydration method. Lipids were dissolved in 1:1 chloroform/methanol, dried to glass vials by evaporation, and kept in vacuum overnight. The dry lipid film was hydrated with PBS, then following repeated heating (50 °C) and cooling (− 196 °C) steps, lipid solutions were extruded through polycarbonate filters with 200 nm pore size (at least 11 times) using a LIPEX extruder (Northem Lipids Inc., Canada). Final lipid concentration was 13 mM. For mimicking mammalian and bacterial cell membrane, liposomes of pure DOPC and DOPC/DOPG (80/20 n/n%) referred to as PC and PC/PG, respectively, were used throughout in the study.

### Assay conditions

To mimic physiological conditions, all biophysical experiments were carried out in phosphate-buffered saline (PBS, 10 mM phosphate, 137 mM NaCl, and 3 mM KCl, pH 7.4). For the three-component systems, mixtures differed in the mixing order. Two components, shown first in parentheses, were preincubated, followed by the addition of the third component, e.g. (peptide + small molecule) + liposome.

### Circular dichroism spectroscopy

Spectra were collected using a JASCO J-1500 spectropolarimeter at room temperature in PBS. Spectra were recorded in the far-UV region (195–250 nm) at a speed of 50 nm/min with a bandwidth of 1 nm using a cylindrical, quartz cuvette of 1 mm path-length. Spectra were corrected by subtracting a matching blank (buffer or lipid containing buffer), and smoothed. 25 µM peptide was titrated with molar equivalents of hemin or biliverdin in the presence and absence of PC or PC/PG liposomes. Note that for samples containing liposomes and/or higher aggregates, signals at low wavelengths are not reliable due to light scattering, thus these regions were truncated in the corresponding figures.

### Attenuated total reflectance Fourier-transform infrared (ATR-FTIR) spectrometry

ATR-FTIR spectra were acquired using a Varian 2000 FTIR Scimitar Series spectrometer fitted with a *Golden Gate* single reflection diamond ATR accessory (Specac Ltd, UK). 5 μl of sample was mounted on the diamond ATR crystal. The measurements were done after drying the sample by slow evaporation of the solvent buffer under ambient conditions. 64 scans were co-added at a nominal resolution of 2 cm^−1^. The GRAMS/32 software package (Galactic Inc, USA) was used for all spectral manipulations.

### Dynamic light scattering

Particle size and size distribution of the peptide-small molecule mixtures were measured at 20 °C using a W130i dynamic light scattering device (DLS, Avid Nano Ltd., High Wycombe, UK) with a diode laser (660 nm) and a photodiode detector. Low volume disposable cuvettes with 1 cm path-length were used (UVette, Eppendorf Austria GmbH). Samples prepared for CD experiments were measured after completing the titration. The time-dependent autocorrelation function was measured for 10 s, repeated 10 times.

### Transmission electron microscopy

For direct visualisation of the structure and morphology of the sample, transmission electron microscopy images were obtained. The precipitate of the peptide-hemin mixture prepared in PBS was resuspended in MQ water then a 2 μl droplet was mounted onto a 200 mesh copper grid with a support film made of formvar and stained with phosphotungstic acid or uranyl acetate. Measurements were performed in a JEOL JEM 1011 transmission electron microscope operating at 80 kV, and images were taken with Olympus Morada 11-megapixel camera and iTEM software (Olympus).

### Fluorescence spectroscopy

Spectra were collected using a Jobin Yvon Fluoromax-3 spectrofluorometer at 25 °C in PBS. Spectra were recorded three times, averaged, and corrected by subtracting an appropriate blank. The Trp fluorophore was excited at 295 nm, and emission was monitored from 305 to 400 nm. 2 µM peptide was titrated with subsequent addition of hemin (up to 20 μM) in the presence and absence of liposomes (100 µM total lipid).

### Linear dichroism spectroscopy

Linear dichroism measurements were performed on a JASCO-1500 spectropolarimeter equipped with a Couette flow cell unit at 25 °C in PBS supplemented with 50 w% sucrose. Spectra were recorded between 200 and 500 nm at a rate of 100 nm/min, with a data pitch of 0.5 nm, response time of 1 s, bandwidth of 1 nm, and a total path length of 0.5 mm. Liposome samples (with a total lipid concentration of 1.3 mM) were oriented under a shear gradient of 2270 s^−1^, and spectra measured at zero shear gradient were subtracted. The concentration of CM15 and LL-37 was 85 µM and 50 µM, respectively, and peptide to hemin molar ratio was 1:3 with both peptides. Absorbance spectra were obtained by direct conversion of the recorded HT data.

### Cell culturing and cytostasis assays

EBC-1 (squamous cell carcinoma, metastatic, origin: bronchi, JCRB No: JCRB0820^85,86^, originally purchased from Thermo Fisher Scientific and provided by the National Institute of Oncology, Budapest, Hungary) cells were maintained as an adherent culture in DMEM medium (Lonza) containing 10% FBS (Gibco) and supplemented with 2 mM of l-glutamine (Lonza), 1% non-essential amino acids (Gibco), 1 mM sodium pyruvate (Sigma-Aldrich), 1% penicillin–streptomycin (10,000 units penicillin and 10 mg streptomycin/ml, Gibco), at 37 °C in a humidified atmosphere containing 5% CO_2_.

For testing cytostatic effects, 3500 cells per well were plated in 96-well flat bottom tissue culture plates (Sarstedt, Nümbrecht, Germany) in complete DMEM medium. LL-37 stock solution was prepared in serum-free medium (SFM) at 200 µM, while heme compounds were dissolved first in 10 mM NaOH at 3 mM, sterile filtered, and diluted tenfold in SFM. Cells were incubated with the peptide, small molecules, or peptide-small molecule mixtures for 24 h. Then cells were washed three times with SFM and cultured further for 72 h in complete medium. Cytostatic activity was determined using the Alamar Blue assay, where 20 µl Alamar Blue (resazurin sodium salt, Sigma-Aldrich) solution (0.15 mg/ml, dissolved in PBS, pH 7.4) was added to each well. Following a 3 h incubation, fluorescence (λEx = 530/30 and λEm = 610/10 nm) was read using a Synergy H4 multi-mode microplate reader (BioTek, Winooski, VT, USA). Live cell percentage of treated cells was calculated compared to control cells treated with medium only. All measurements were performed in triplicates, and live cell % values together with SEM are presented.

### Cell morphology

To visualize cell morphology changes, microscopic images of EBC-1 cells were captured. EBC-1 cells were plated in a 96-well flat bottom tissue culture plate and treated with peptide, heme compounds, and their mixtures as described in the above section. Microscopic images of the adherent cells were taken after the first 3 h of the treatment using an Olympus CKX41 microscope (Hamburg, Germany, objective: 20×).

## Supplementary Information

Supplementary Information.

## Data Availability

Data generated or analysed during this study are included in this article and its Supplementary Information file and are available from the corresponding authors upon request.

## References

[1] Orf K, Cunnington A (2015). Infection-related hemolysis and susceptibility to Gram-negative bacterial co-infection. Front. Microbiol..

[2] Martins R (2016). Heme drives hemolysis-induced susceptibility to infection via disruption of phagocyte functions. Nat. Immunol..

[3] Nguyen LT, Haney EF, Vogel HJ (2011). The expanding scope of antimicrobial peptide structures and their modes of action. Trends Biotechnol..

[4] Zasloff M (2002). Antimicrobial peptides of multicellular organisms. Nature.

[5] Di Somma A, Moretta A, Canè C, Cirillo A, Duilio A (2020). Antimicrobial and antibiofilm peptides. Biomolecules.

[6] Khamis AM, Essack M, Gao X, Bajic VB (2015). Distinct profiling of antimicrobial peptide families. Bioinformatics.

[7] Brown KL, Hancock RE (2006). Cationic host defense (antimicrobial) peptides. Curr. Opin. Immunol..

[8] Pirtskhalava M (2020). DBAASP v3: Database of antimicrobial/cytotoxic activity and structure of peptides as a resource for development of new therapeutics. Nucleic Acids Res..

[9] Haney EF, Straus SK, Hancock RE (2019). Reassessing the host defense peptide landscape. Front. Chem..

[10] Brogden KA (2005). Antimicrobial peptides: Pore formers or metabolic inhibitors in bacteria?. Nat. Rev. Microbiol..

[11] Bals R, Wilson JM (2003). Cathelicidins—A family of multifunctional antimicrobial peptides. Cell Mol. Life Sci..

[12] Hancock RE, Haney EF, Gill EE (2016). The immunology of host defence peptides: Beyond antimicrobial activity. Nat. Rev. Immunol..

[13] Sani MA, Separovic F (2016). How membrane-active peptides get into lipid membranes. Acc. Chem. Res..

[14] Hollmann A, Martinez M, Maturana P, Semorile LC, Maffia PC (2018). Antimicrobial peptides: Interaction with model and biological membranes and synergism with chemical antibiotics. Front. Chem..

[15] Hollmann A (2016). Role of amphipathicity and hydrophobicity in the balance between hemolysis and peptide–membrane interactions of three related antimicrobial peptides. Colloids Surf. B.

[16] Melo MN, Ferre R, Castanho MA (2009). Antimicrobial peptides: Linking partition, activity and high membrane-bound concentrations. Nat. Rev. Microbiol..

[17] Zsila F, Beke-Somfai T (2020). Human host-defense peptide LL-37 targets stealth siderophores. Biochem. Biophys. Res. Commun..

[18] Zsila F, Bősze S, Horváti K, Szigyártó IC, Beke-Somfai T (2017). Drug and dye binding induced folding of the intrinsically disordered antimicrobial peptide CM15. RSC Adv..

[19] Zsila F, Juhasz T, Bosze S, Horvati K, Beke-Somfai T (2017). Hemin and bile pigments are the secondary structure regulators of intrinsically disordered antimicrobial peptides. Chirality.

[20] Zsila F, Kohut G, Beke-Somfai T (2019). Disorder-to-helix conformational conversion of the human immunomodulatory peptide LL-37 induced by antiinflammatory drugs, food dyes and some metabolites. Int. J. Biol. Macromol..

[21] Queme-Pena M (2019). Manipulating active structure and function of cationic antimicrobial peptide CM15 with the polysulfonated drug suramin: A step closer to in vivo complexity. ChemBioChem.

[22] Quemé-Peña M (2020). Old polyanionic drug suramin suppresses detrimental cytotoxicity of the host defense peptide LL-37. ACS Pharmacol. Transl. Sci..

[23] Ricci M (2020). Anionic food color tartrazine enhances antibacterial efficacy of histatin-derived peptide DHVAR4 by fine-tuning its membrane activity. Q. Rev. Biophys..

[24] He J, Starr CG, Wimley WC (2015). A lack of synergy between membrane-permeabilizing cationic antimicrobial peptides and conventional antibiotics. Biochim. Biophys. Acta.

[25] Burmester T, Hankeln T (2014). Function and evolution of vertebrate globins. Acta Physiol. (Oxf.).

[26] Poulos TL (2014). Heme enzyme structure and function. Chem. Rev..

[27] Shimizu T, Lengalova A, Martinek V, Martinkova M (2019). Heme: emergent roles of heme in signal transduction, functional regulation and as catalytic centres. Chem. Soc. Rev..

[28] Bozza MT, Jeney V (2020). Pro-inflammatory actions of heme and other hemoglobin-derived DAMPS. Front. Immunol..

[29] Graca-Souza AV, Arruda MA, de Freitas MS, Barja-Fidalgo C, Oliveira PL (2002). Neutrophil activation by heme: Implications for inflammatory processes. Blood.

[30] Robinson SR, Dang TN, Dringen R, Bishop GM (2009). Hemin toxicity: A preventable source of brain damage following hemorrhagic stroke. Redox Rep..

[31] Kumar S, Bandyopadhyay U (2005). Free heme toxicity and its detoxification systems in human. Toxicol. Lett..

[32] Lim EJ (2010). Hemin inhibits cyclin D1 and IGF-1 expression via STAT5b under hypoxia in ERalpha-negative MDA-MB 231 breast cancer cells. Int. J. Oncol..

[33] Schmitt TH, Frezzatti WA, Schreier S (1993). Hemin-induced lipid membrane disorder and increased permeability: A molecular model for the mechanism of cell lysis. Arch. Biochem. Biophys..

[34] Mateus V, Rocha J, Mota-Filipe H, Sepodes B, Pinto R (2018). Hemin reduces inflammation associated with TNBS-induced colitis. Clin. Exp. Gastroenterol..

[35] Belcher JD, Nath KA, Vercellotti GM (2013). Vasculotoxic and proinflammatory effects of plasma heme: Cell signaling and cytoprotective responses. ISRN Oxid. Med..

[36] Cho JH, Sung BH, Kim SC (2009). Buforins: Histone H2A-derived antimicrobial peptides from toad stomach. Biochim. Biophys. Acta.

[37] Vandamme D, Landuyt B, Luyten W, Schoofs L (2012). A comprehensive summary of LL-37, the factotum human cathelicidin peptide. Cell. Immunol..

[38] Wang G, Matsuzaki K (2019). Design of antimicrobial peptides: Progress made with human cathelicidin LL-37. Antimicrobial Peptides.

[39] Kim HS (2000). Pepsin-mediated processing of the cytoplasmic histone H2A to strong antimicrobial peptide buforin I. J. Immunol..

[40] Ruissen AL (2001). Effects of histatin 5 and derived peptides on *Candida albicans*. Biochem. J..

[41] Palma MS (2006). Handbook of Biologically Active Peptides.

[42] Kastin A (2013). Handbook of Biologically Active Peptides.

[43] Martin I, Goormaghtigh E, Ruysschaert J-M (2003). Attenuated total reflection IR spectroscopy as a tool to investigate the orientation and tertiary structure changes in fusion proteins. Biochim. Biophys. Acta.

[44] Barth A (2007). Infrared spectroscopy of proteins. Biochim. Biophys. Acta.

[45] Terwilliger TC, Weissman L, Eisenberg D (1982). The structure of melittin in the form I crystals and its implication for Melittin’s lytic and surface activities. Biophys. J..

[46] Ishida KP, Griffiths PR (1993). Comparison of the amide I/II intensity ratio of solution and solid-state proteins sampled by transmission, attenuated total reflectance, and diffuse reflectance spectrometry. Appl. Spectrosc..

[47] Ganguly D (2009). Self-RNA-antimicrobial peptide complexes activate human dendritic cells through TLR7 and TLR8. J. Exp. Med..

[48] Engelberg Y, Landau M (2020). The Human LL-37 (17–29) antimicrobial peptide reveals a functional supramolecular structure. Nat. Commun..

[49] Kurgan KW (2019). Retention of native quaternary structure in racemic melittin crystals. J. Am. Chem. Soc..

[50] Tosteson MT, Holmes SJ, Razin M, Tosteson DC (1985). Melittin lysis of red cells. J. Membr. Biol..

[51] Milani A, Benedusi M, Aquila M, Rispoli G (2009). Pore forming properties of cecropin-melittin hybrid peptide in a natural membrane. Molecules.

[52] Nordén B, Rodger A, Dafforn T (2019). Linear Dichroism and Circular Dichroism: A Textbook on Polarized-Light Spectroscopy.

[53] Caesar CE, Esbjorner EK, Lincoln P, Norden B (2009). Assigning membrane binding geometry of cytochrome C by polarized light spectroscopy. Biophys. J..

[54] Gamiz-Arco G (2021). Heme-binding enables allosteric modulation in an ancient TIM-barrel glycosidase. Nat. Commun..

[55] Inamura I, Isshiki M, Araki T (1989). Solubilization of hemin in neutral and acidic aqueous solutions by forming complexes with water-soluble macromolecules. Bull. Chem. Soc. Jpn..

[56] Inada Y, Shibata K (1962). The Soret band of monomeric hematin and its changes on polymerization. Biochem. Biophys. Res. Commun..

[57] Sohoni S (2019). Elevated heme synthesis and uptake underpin intensified oxidative metabolism and tumorigenic functions in non-small cell lung cancer cells. Cancer Res..

[58] Kosciuczuk EM (2012). Cathelicidins: Family of antimicrobial peptides. A review. Mol. Biol. Rep..

[59] Gemelli C, Dongmo BM, Ferrarini F, Grande A, Corsi L (2014). Cytotoxic effect of hemin in colonic epithelial cell line: Involvement of 18 kDa translocator protein (TSPO). Life Sci..

[60] Higdon AN (2012). Hemin causes mitochondrial dysfunction in endothelial cells through promoting lipid peroxidation: The protective role of autophagy. Am. J. Physiol.-Heart Circ. Physiol..

[61] Utsugi T, Schroit AJ, Connor J, Bucana CD, Fidler IJ (1991). Elevated expression of phosphatidylserine in the outer membrane leaflet of human tumor cells and recognition by activated human blood monocytes. Can. Res..

[62] Jia X, Gu Z, Chen W, Jiao J (2016). Tigecycline targets nonsmall cell lung cancer through inhibition of mitochondrial function. Fundam. Clin. Pharmacol..

[63] Horváti K (2017). Comparative analysis of internalisation, haemolytic, cytotoxic and antibacterial effect of membrane-active cationic peptides: Aspects of experimental setup. Amino Acids.

[64] Roshanak S, Shahidi F, Yazdi FT, Javadmanesh A, Movaffagh J (2020). Evaluation of antimicrobial activity of Buforin I and Nisin and the synergistic effect of their combination as a novel antimicrobial preservative. J. Food Prot..

[65] Čeřovský V (2009). Lasioglossins: Three novel antimicrobial peptides from the venom of the eusocial bee *Lasioglossum laticeps* (Hymenoptera: Halictidae). ChemBioChem.

[66] Slaninová J (2012). Toxicity study of antimicrobial peptides from wild bee venom and their analogs toward mammalian normal and cancer cells. Peptides.

[67] Zhao R (2009). Molecular cloning of two novel temporins from *Lithobates catesbeianus* and studying of their antimicrobial mechanisms. Prog. Biochem. Biophys..

[68] Gunasekera S, Muhammad T, Strömstedt AA, Rosengren KJ, Göransson U (2020). Backbone cyclization and dimerization of LL-37-derived peptides enhance antimicrobial activity and proteolytic stability. Front. Microbiol..

[69] Mohammed I, Said DG, Nubile M, Mastropasqua L, Dua HS (2019). Cathelicidin-derived synthetic peptide improves therapeutic potential of vancomycin against *Pseudomonas aeruginosa*. Front. Microbiol..

[70] Wißbrock A, Paul George AA, Brewitz HH, Kühl T, Imhof D (2019). The molecular basis of transient heme-protein interactions: Analysis, concept and implementation. Front. Microbiol..

[71] Robertson DE (1994). Design and synthesis of multi-haem proteins. Nature.

[72] Choma CT (1994). Design of a heme-binding four-helix bundle. J. Am. Chem. Soc..

[73] Mahajan M, Bhattacharjya S (2014). Designed di-heme binding helical transmembrane protein. ChemBioChem.

[74] D’Souza A, Bhattacharjya S (2021). De novo-designed β-sheet heme proteins. Biochemistry.

[75] D'Souza A, Wu X, Yeow EKL, Bhattacharjya S (2017). Designed heme-cage β-sheet miniproteins. Angew. Chem..

[76] Mahajan M, Bhattacharjya S (2013). β-hairpin peptides: Heme binding, catalysis, and structure in detergent micelles. Angew. Chem..

[77] Kohut G (2019). The molecular mechanism of structural changes in the antimicrobial peptide CM15 upon complex formation with drug molecule suramin: A computational analysis. Phys. Chem. Chem. Phys..

[78] Hale JD, Hancock RE (2007). Alternative mechanisms of action of cationic antimicrobial peptides on bacteria. Expert Rev. Anti Infect. Ther..

[79] Kohut G, Juhász TN, Quemé-Peña M, Bősze SE, Beke-Somfai T (2021). Controlling peptide function by directed assembly formation: Mechanistic insights using multiscale modeling on an antimicrobial peptide–drug–membrane system. ACS Omega.

[80] Oh J-Y (2016). Absorbance and redox based approaches for measuring free heme and free hemoglobin in biological matrices. Redox Biol..

[81] Blyth GA, Connors L, Fodor C, Cobo ER (2020). The network of colonic host defense peptides as an innate immune defense against enteropathogenic bacteria. Front. Immunol..

[82] Costa DL, Amaral EP, Andrade BB, Sher A (2020). Modulation of inflammation and immune responses by heme oxygenase-1: Implications for infection with intracellular pathogens. Antioxidants.

[83] Wandersman C, Delepelaire P (2004). Bacterial iron sources: From siderophores to hemophores. Annu. Rev. Microbiol..

[84] Schaer DJ, Vinchi F, Ingoglia G, Tolosano E, Buehler PW (2014). Haptoglobin, hemopexin, and related defense pathways—Basic science, clinical perspectives, and drug development. Front. Physiol..

[85] Hiraki S (1982). Establishment of human continuous cell lines from squamous cell, adeno-and small cell carcinoma of the lung and the results of heterotransplantation. Haigan.

[86] Imanishi K (1989). Production of transforming growth factor-alpha in human tumour cell lines. Br. J. Cancer.

