# Supplementary material for: Interplay between membrane active host defense peptides and heme modulates their assemblies and in vitro activity
Source: Sci Rep. 2021 Sep 15;11:18328. doi: 10.1038/s41598-021-97779-2 (PMC8443738; doi:10.1038/s41598-021-97779-2)
Supplement: Supplementary file 1 — Supplementary Information. [file 41598_2021_97779_MOESM1_ESM.pdf]

Supplementary Information

Interplay between membrane active host defense peptides and heme modulates their assemblies and *in vitro* activity

Tünde Juhász*[1], Mayra Quemé-Peña[1,2], Bence Kővágó[1], Judith Mihály[1], Maria Ricci[1], Kata Horváti[3,4], Szilvia Bősze[3], Ferenc Zsila[1], Tamás Beke-Somfai*[1]

[1]Institute of Materials and Environmental Chemistry, Research Centre for Natural Sciences, Budapest, Hungary
[2]Hevesy György PhD School of Chemistry, Eötvös Loránd University, Budapest, Hungary
[3]ELKH-ELTE Research Group of Peptide Chemistry, Eötvös Loránd University, Budapest, Hungary
[4]Department of Organic Chemistry, Eötvös Loránd University, Budapest, Hungary

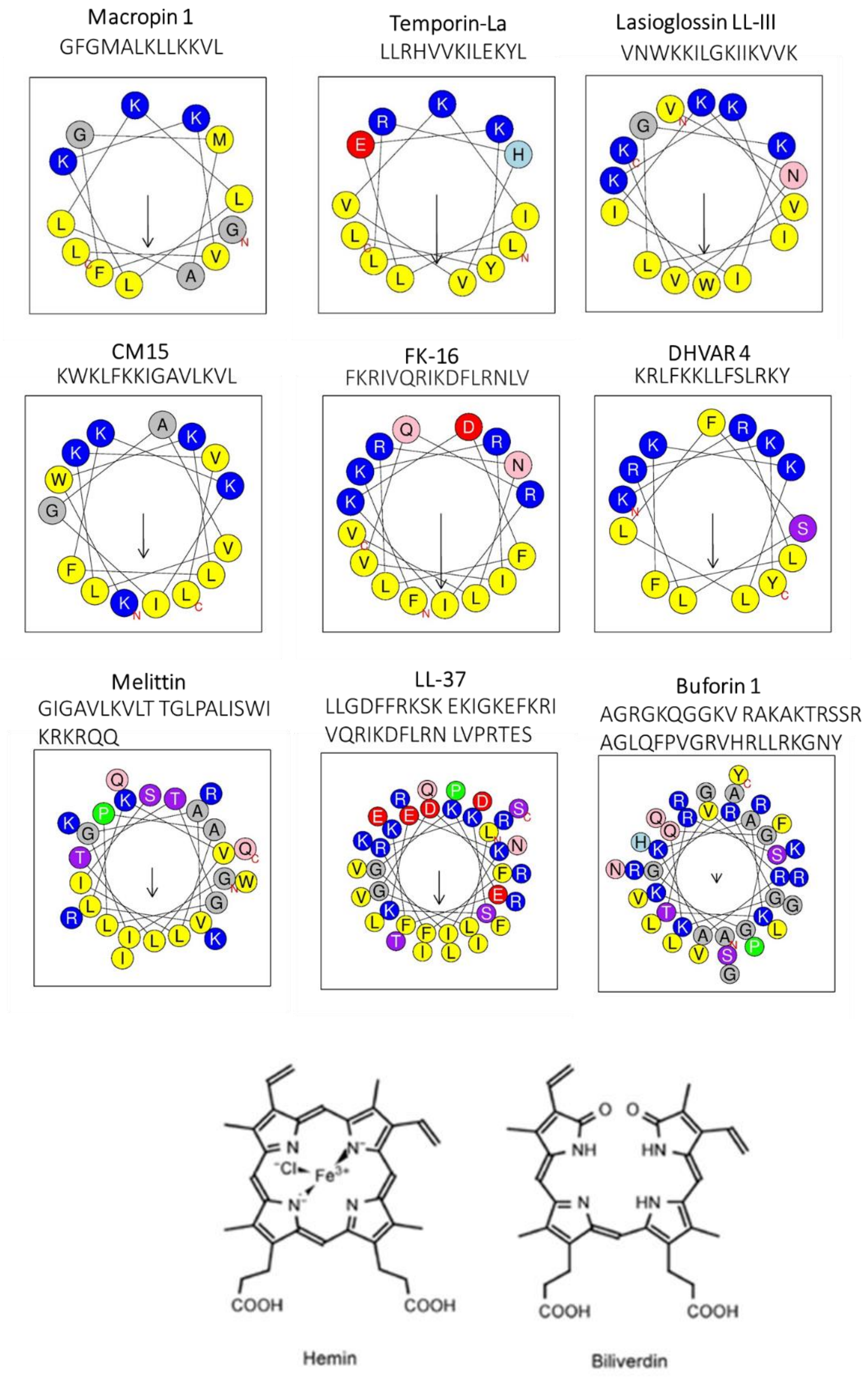

Figure S1. Helical wheel representation of the peptides and chemical structures of the heme derivatives used in the study.

Helical wheel images were drawn with the online tool HeliQuest [1] available at https://heliquest.ipmc.cnrs.fr. Note the good separation of the hydrophobic (yellow) and hydrophilic (positively (blue) or negatively (red) charged, and uncharged polar (purple)) residues. An exception is represented by buforin, for which the separation is poor. In addition, note the high content of negatively charged residues for LL-37, although the peptide is still net positively charged. The arrows indicate the hydrophobic moment. Note the exceptionally small hydrophobic moment for buforin.

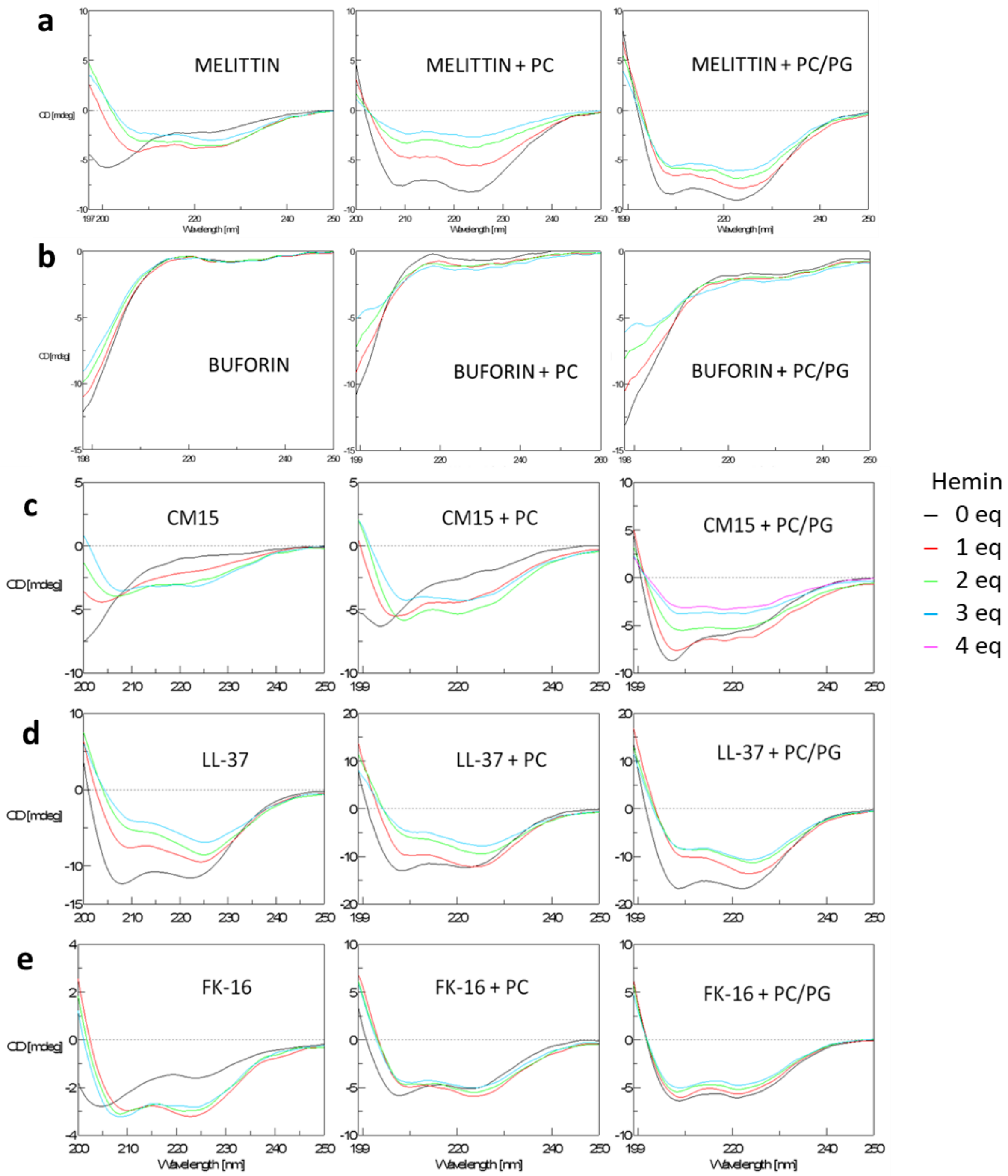

Figure S2. CD spectral changes of selected peptides upon hemin titration in the presence of model membranes. Spectra were collected at 25 μM peptide and 635 μM lipid upon consecutive addition of molar equivalents (eq) of hemin to peptide-lipid mixtures in PBS. For comparison, titrations obtained in the absence of membranes (Figure 1) are also shown.

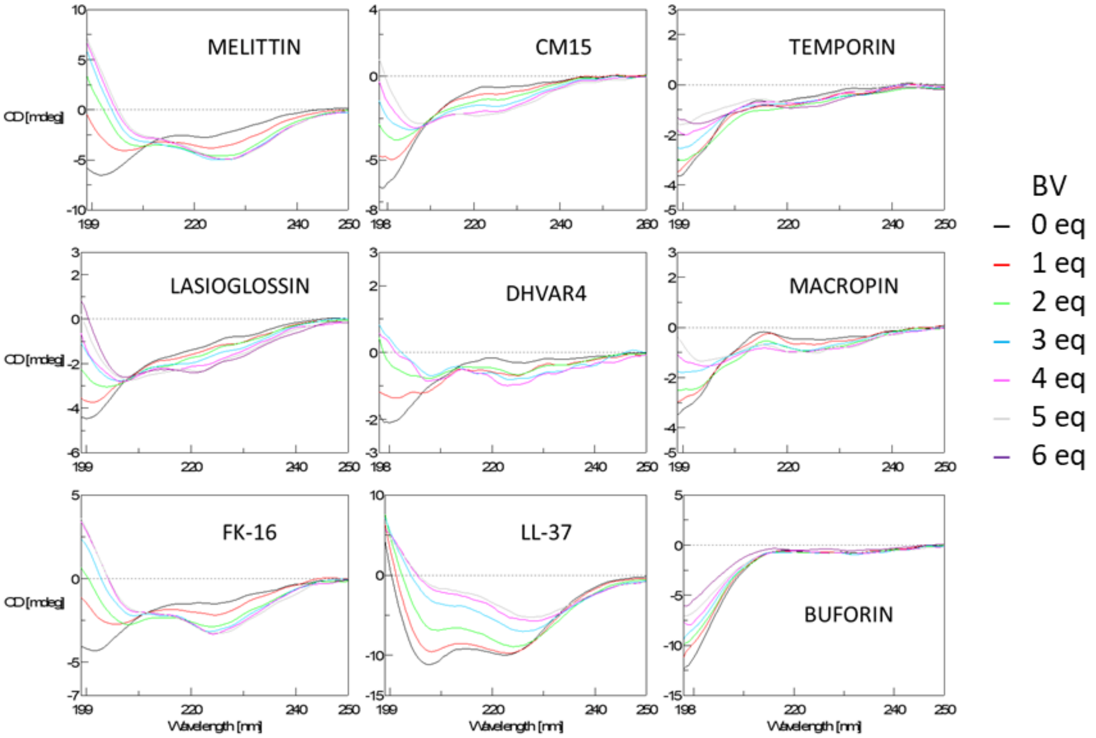

Figure S3. CD spectral changes of the peptides upon titration with biliverdin. Spectra were collected at 25 µM peptide upon consecutive addition of molar equivalents of biliverdin in PBS. Note that ellipticity scales are different.

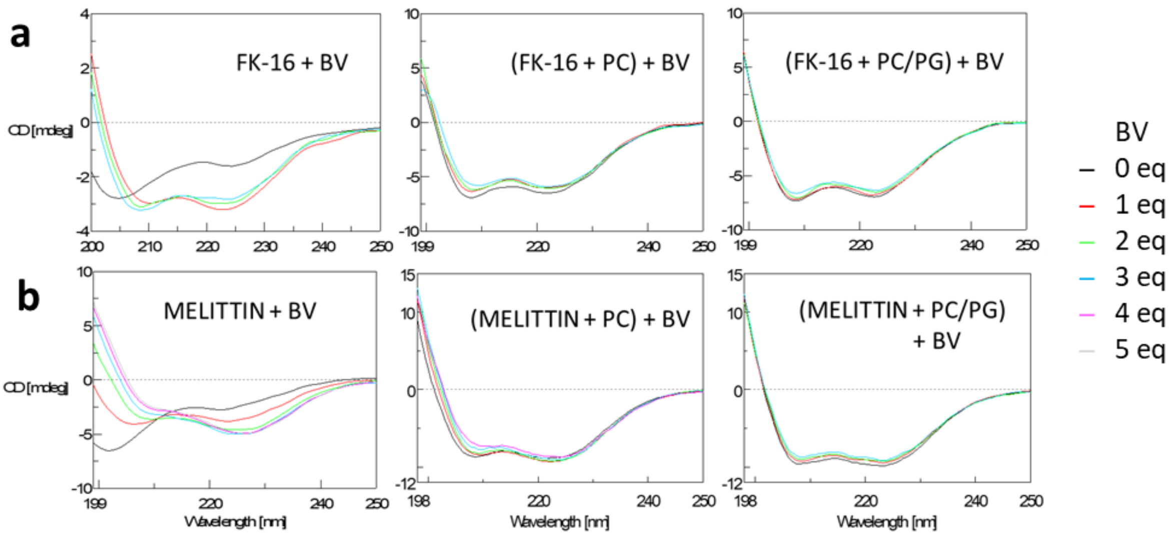

Figure S4. CD spectral changes of selected peptides upon biliverdin titration in the presence of model membranes. Spectra were collected at 25 µM peptide and 635 µM lipid upon consecutive addition of molar equivalents of biliverdin in PBS.

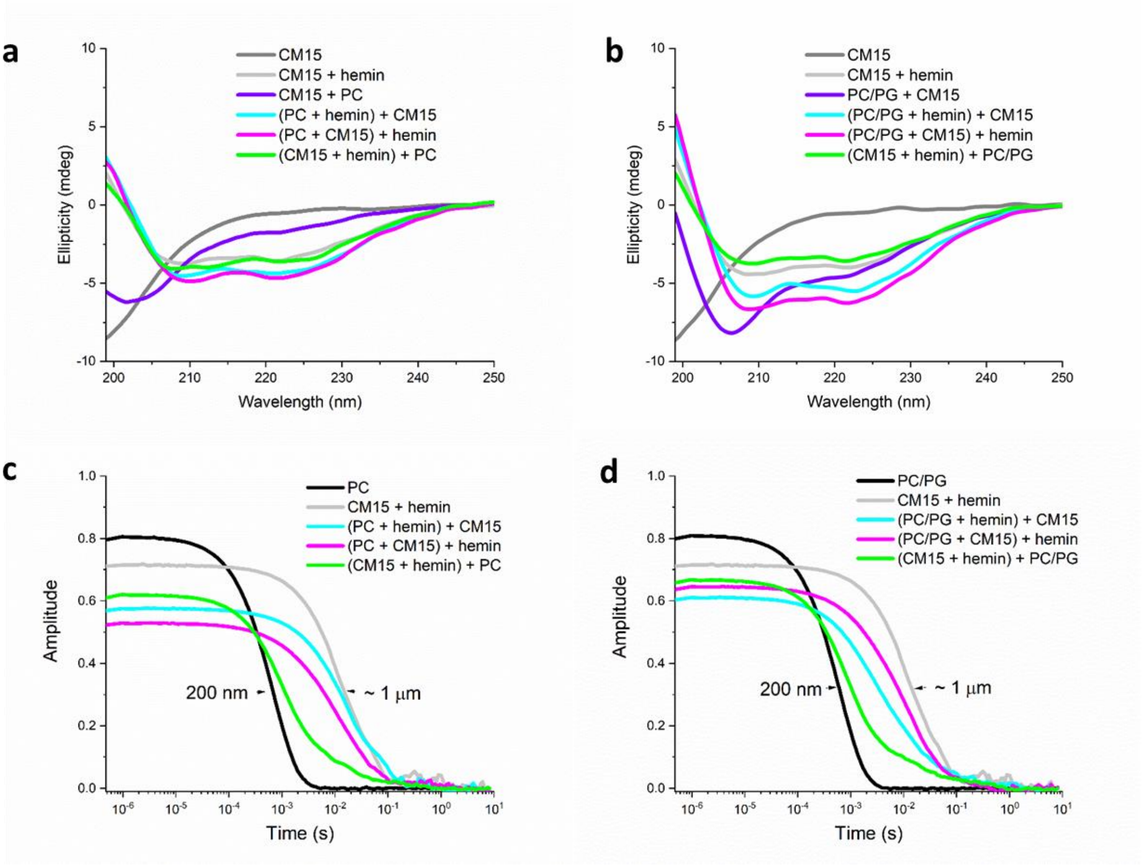

Figure S5. Conformation and partition of CM15 in three-component systems. CD spectra (a,b), and correlation functions (c,d) were recorded at 25 μM peptide, 75 μM hemin and 635 μM lipid in PBS for mixtures with various mixing order of the components. For the correlation functions, the black curves refer to the liposome size of 200 nm while the right-shifted curves are indicative of higher-sized aggregates with a heterogeneous size distribution, where the average particle size is approximately up to ~1 μm.

Table S1. Vibrations analysed in the study.

Vibration	Wavenumber (cm^{-1})	Information obtained
peptide amide I	1600-1500	peptide secondary structure
peptide amide II	1500-1400	peptide assembly
lipid vibrations		
acyl CH_2 str., $\nu_{as}CH_2$ and ν_sCH_2	~2925 and ~2850	lipid order, and packing
ester C=O, νCO	~1735	lipid ester part hydration
phosphate str. $\nu_{as}PO_2^-$	~1245	lipid phosphate hydration
phosphate str. $\nu_sPO_2^-$	~1090	lipid phosphate
phosphate diester str. νR-O-P-O-R'	~1070	lipid phosphate conformation
lipid choline $\nu N(CH_3)_3^+$	~970	lipid choline

str, streching; s, symmetric; as, antisymmetric

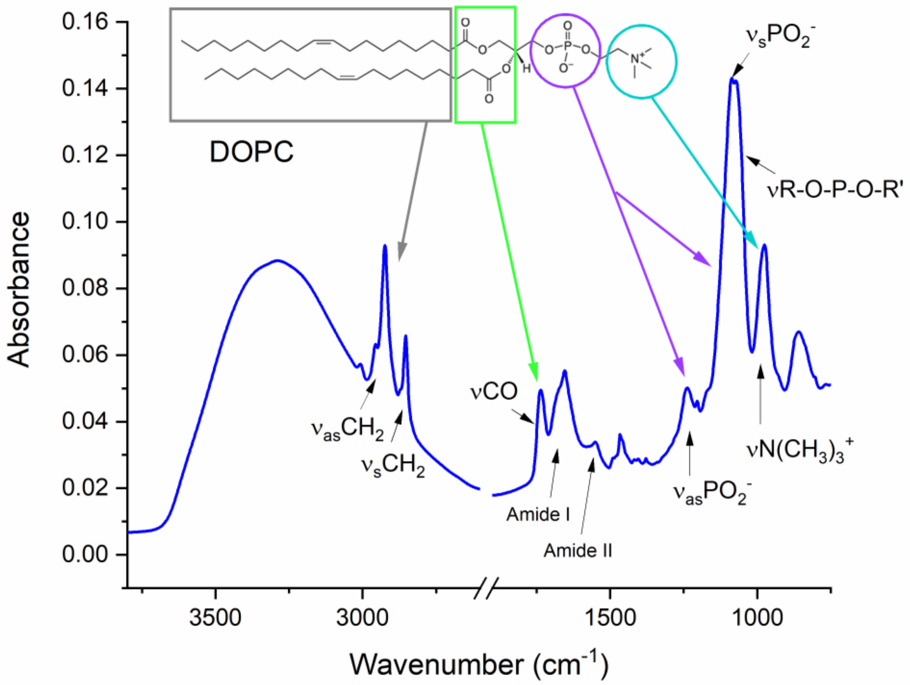

Representative IR spectrum showing the bands used for IR analysis.
Note that bands corresponding to the lipid head-group (phosphate and choline) vibrations overlap with bands originated from the inorganic phosphate moiety of the buffer PBS.

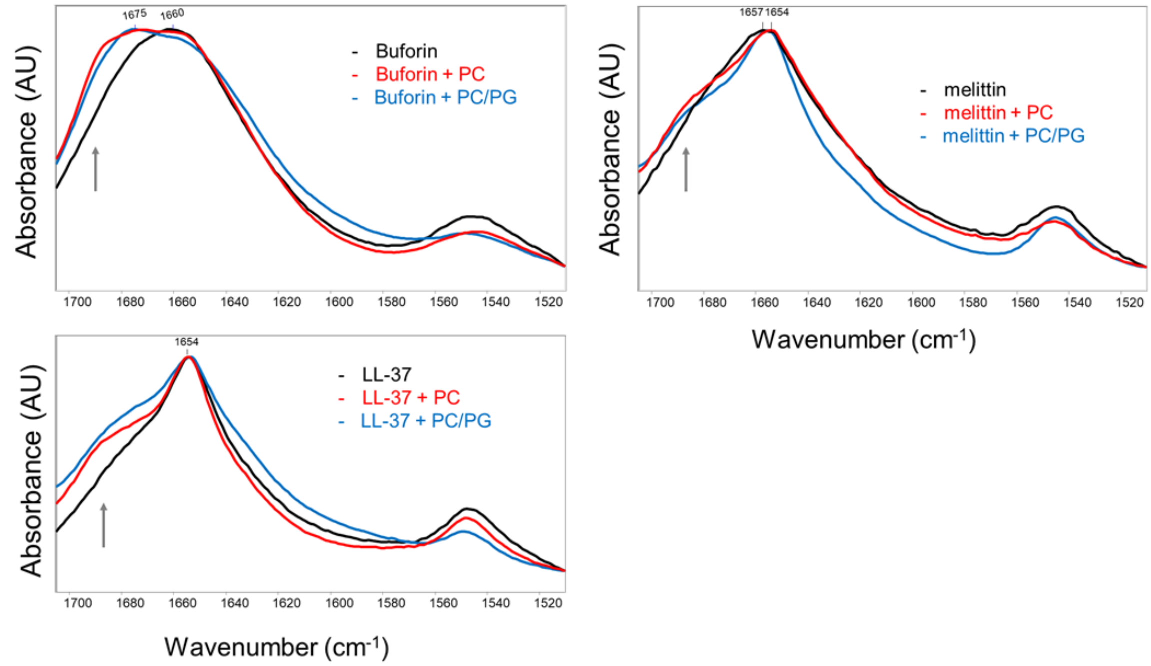

Figure S6. IR analysis: peptide conformational changes upon liposome binding. Amide I and II regions of the spectra are shown collected for dry films obtained from 25 μM peptide solution in the absence of hemin. Spectra are normalized to amide I intensities.

All three peptides investigated showed an extra shoulder at ~1680 cm^{-1} in the Amide I band, indicated by arrows. This spectral feature seems to be common characteristics for the linear cationic amphipathic peptides, it has been already reported for melittin fragments [2] or CM15 [3]. Although the origin of this is not clear, it can presumably be attributed to some rather extended conformation of a peptide region.
As for melittin, many studies focused on its mechanism of action on lipid bilayers in vitro. Results obtained with various techniques were rather controversial, nevertheless, the effect was suggested to depend on the lipid-to-peptide ratio [4], and linked to peptide conformation adopted. In line with these, interpretation of peptide structural changes based on amide I bands remained rather speculative. We performed a comparative IR analysis on the two-component melittin-liposome mixtures (Figure S7). As expected, a clear trend was observed for the peptide amide as well as lipid acyl chain or head group regions upon enhancement of the lipid-to-peptide ratio, *i. e.* keeping the peptide concentration constant (of 50 μM) and increasing the lipid concentration. Combined results are compatible with a binding scenario where higher lipid concentrations allow peptides adopt a more extended conformation on the bilayer surface and this peptide positioning can perturb deeper layers of the lipid bilayer while low lipid levels force the bound peptides into a more compact conformation leading to peptide accumulation still on the membrane surface, where tight peptide packing precludes their deep insertion into the membrane. In contrast, peptide binding to the membrane resulted in more ordered lipid chains with tighter packing for melittin, where the most remarkable effect was observed when hemin was added to the PC/PG liposome-bound peptide. However, no significant shift in the lipid C=O band indicated no perturbation of the bilayer at the hydrophobic-hydrophilic boundary. Nevertheless, changes in the phosphate vibrations indicated variations in lipid head group-conformation, which could likely compensate for changes in acyl chain packing. Thus, the effect might be attributed to anchoring tryptophan (Trp) residue of melittin. As the Trp indole ring commonly points outwards almost perpendicular to the helix axis [5], in a surface-associated peptide orientation, the Trp side chain would be inserted among the lipid acyl chains, resulting in turn in lipid packing of a more ordered fashion. In contrast, surface positioned peptides lacking rigid anchoring residues can disturb the intrinsic membrane structure by bending preferentially the lipid headgroups while pushing deeper membrane layers decreasing their order as observed for buforin or LL-37. Similar effect was observed for hemin binding to liposomes (Figure S11) indicating that similar perturbation can be induced by membrane active molecules of different nature.

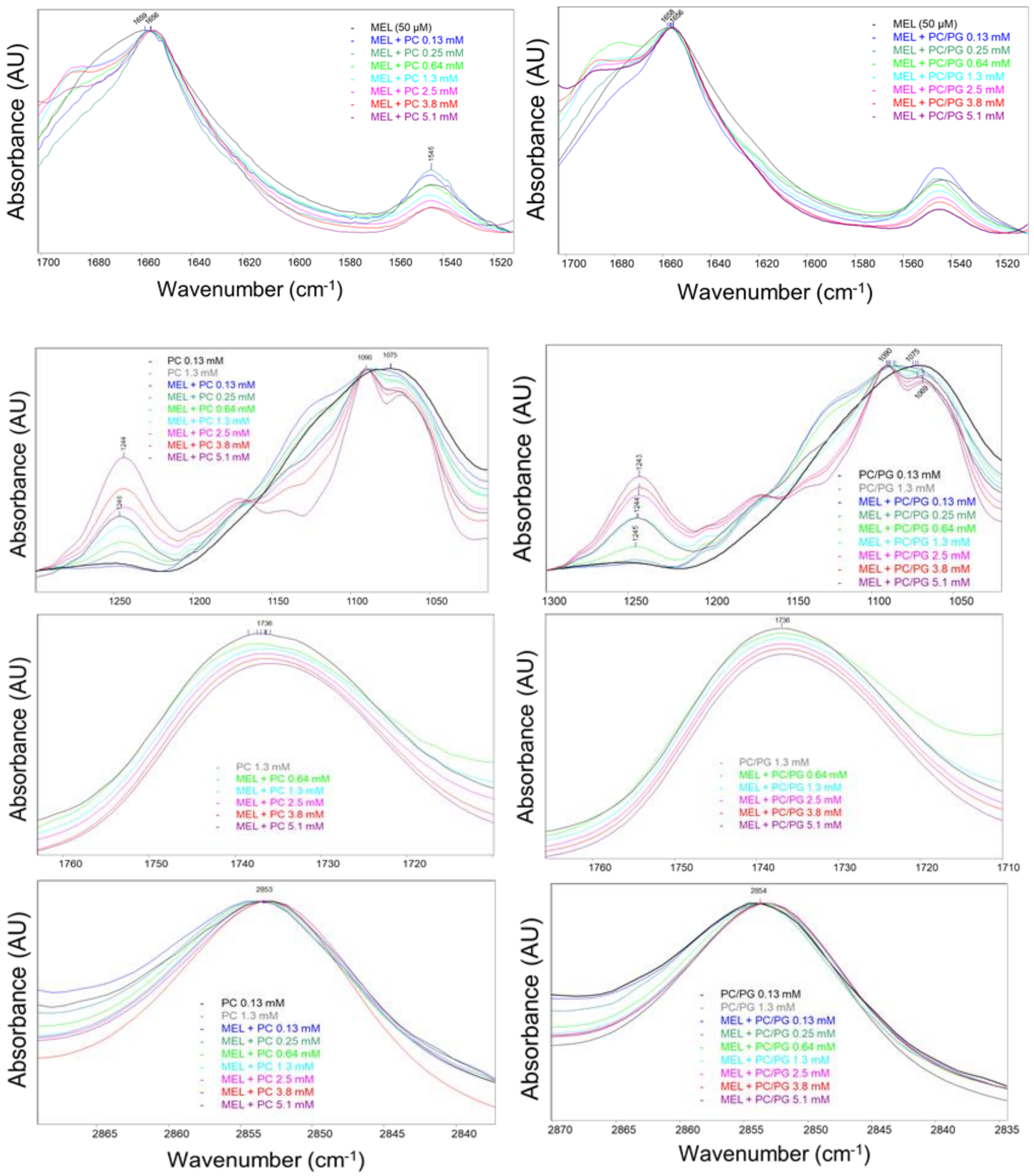

Figure S7. IR analysis of the melittin-lipid interaction: Peptide conformational changes upon lipid binding and peptide-induced perturbation of the lipid bilayers depending on the lipid to peptide ratio.

Conclusions derived for the melittin-lipid interaction:

- most ordered peptide, and highest peptide association at low lipid to peptide ratios with both PC and PC/PG.
- lipid head-group phosphate is bent at all lipid to peptide ratios, most at highest ratios, with both PC and PC/PG.
- peptide is H-bonded to lipid head-group phosphate at all lipid to peptide ratios but only with PC not with PC/PG.
- peptide interacts with the lipid neck only with PC at high lipid to peptide ratios.

peptide induces tighter lipid packing and more ordered lipid chains with both PC and PC/PG, were the higher is the lipid to peptide ratio the higher is the effect.

Table S2. Peptide partition in the peptide-hemin-lipid systems based on analysis of the Amide I and Amide II bands. Peptide partition order in the three-component mixtures was evaluated bason on two characteristic spectral features depicted in Figure 5, i) the lipid-bound fraction corresponding to the amide I component at ~1680 cm^{-1}, ii) and the hemin-bound fraction corresponding to the increased relative amide II band intensity.

Mixture	Peptide state	Binding order
BUF+ PC	Lipid-bound	Pep+lip>(lip+hem)+pep>(pep+hem)+lip>(lip+pep)+hem>pep+hem
	Hemin-bound	Pep+hem>(lip+pep)+hem>(lip+hem)+pep~(pep+hem)+lip>pep+lip
LL-37 + PC	Lipid-bound	Pep+lip>(lip+hem)+pep~(pep+hem)+lip>(lip+pep)+hem>pep+hem
	Hemin-bound	Pep+hem>(lip+pep)+hem~(pep+hem)+lip>(lip+hem)+pep>pep+lip
BUF+ PC/PG	Lipid-bound	Pep+lip>(lip+pep)+hem~(lip+hem)+pep>(pep+hem)+lip>pep+hem
	Hemin-bound	Pep+hem>(pep+hem)+lip>(lip+hem)+pep~(lip+pep)+hem>pep+lip
LL-37 + PC/PG	Lipid-bound	Pep+lip~(lip+pep)+hem~(lip+hem)+pep>(pep+hem)+lip>pep+hem
	Hemin-bound	Pep+hem>(pep+hem)+lip>(lip+hem)+pep~(lip+pep)+hem>pep+lip

pep, peptide; hem, hemin; lip, lipid/liposome

Peptide-induced perturbation of the lipid bilayers

The peptide-hemin-lipid interaction network was further characterized analysing lipid vibrations corresponding to the acyl chain, the ester neck, and head-group phosphate or choline (Table S1) using LL-37 and buforin.

Variations in phosphate vibrations (Figure S8, Table S3) were observed for each mixture investigated, which can be attributed to peptide-induced changes in the lipid head-group region. The nature of the perturbation was dependent on the particular peptide rather than the lipid composition, although the magnitude of the effect was influenced by the latter too. This was well illustrated on buforin where perturbations were detected for all hemin-containing mixtures but more significantly with PC/PG over PC. Spectral changes in the two main phosphate vibrations allowed further differentiation. For the LL-37 – PC/PG system, data are compatible with a binding mode of the peptide in the three-component mixtures where the bending of the head-group remained unchanged while the H-bonding pattern around the phosphate group varied. In contrast, both changed in the two-component PC/PG - LL-37 system. All these indicated that the peptide is immersed deeper into the bilayer when no hemin was present. However, shifts in the lipid ester bands (Figure S9, Table S4) for the lipid-bound peptide, and also for the three-component systems indicated that both hydration layers, *i. e.* the outer one around the head-group phosphate and the inner one around the lipid neck, are affected in these cases.

Analysis of the lipid ester C=O vibrations (Figure S9, Table S4) yielded information on the lipid-binding mode of buforin. Based on the results, this peptide responds to the membrane matrix in a unique way, demonstrating that peptides forming no classical regular amphipathic helix Nevertheless, IR analysis revealed that even buforin could adopt a more ordered conformation could also affect the integrity of lipid bilayers significantly. The rightward shift of the lipid ester C=O vibration and band shape variations, due to changes in the hydrated state of the region, indicated binding of the peptide down to the lipid "neck" part. Similar effects were observed for the buforin-hemin-lipid mixtures suggesting that the peptide remains bound to the vesicles even in the presence of hemin. Buforin also affected the lipid acyl chain vibrations (Figure S10, Table S5), which effect revailed in the presence of hemin. However, the direction of the shift was opposite when the preformed buforin-hemin complex was added to PC/PG, indicating a different binding mode in the latter case, presumably upon association of the hemin-complexed peptide to the membrane.

Based on results on the C-H vibrations (Figure S10, Table S5), membrane interaction with buforin and LL-37 perturbed the lipid order marginally, although small shifts to higher wavenumbers indicated some loosening in lipid packing.

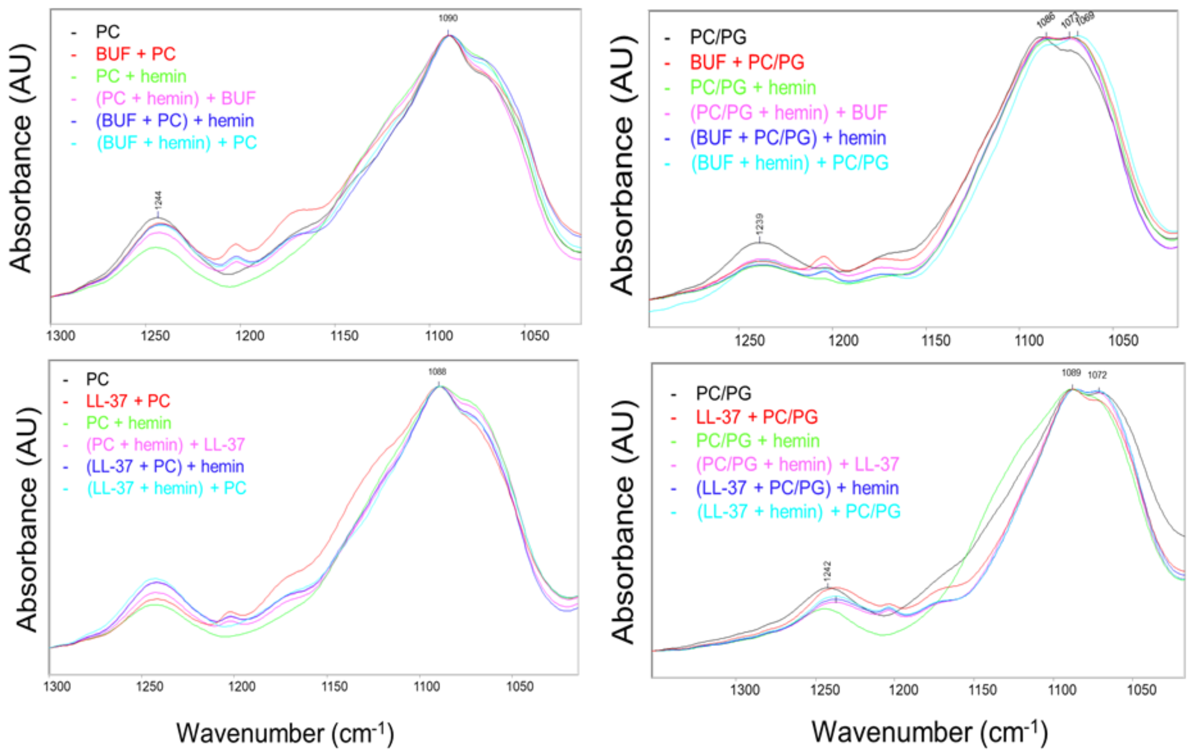

Figure S8. IR analysis on the peptide-induced perturbation of the lipid bilayers: head-group phosphate region. Significant variations observed are listed in the Table below.

Table S3. IR analysis on the peptide-induced perturbation of the lipid bilayers: head-group phosphate region. Significant variations observed for the phosphate vibrations in the spectra shown above.

Mixture	Vibration	Lipid perturbation
BUF + PC	$\nu_{as}PO_2^-$	all →
	νR-O-P-O-R' / $\nu_sPO_2^-$	(pep+lip)+hem ↑, (pep+hem)+lip ↑
LL-37 + PC	$\nu_{as}PO_2^-$	(lip+hem)+pep →, pep+lip →, (pep+hem)+lip ←
	νR-O-P-O-R' / $\nu_sPO_2^-$	(lip+hem)+pep ↑, pep+lip ↓
BUF+ PC/PG	$\nu_{as}PO_2^-$	all →, most (pep+hem)+lip
	νR-O-P-O-R' / $\nu_sPO_2^-$	all ↑, most (pep+hem)+lip
LL-37 + PC/PG	$\nu_{as}PO_2^-$	all →
	νR-O-P-O-R' / $\nu_sPO_2^-$	pep+lip ↓

← or →, right or red shift of the band maximum compared to the sole lipid
↑ or ↓, increase or decrease in the relative intensity of the band varied, compared to the sole lipid
pep, peptide; hem, hemin; lip, lipid/liposome

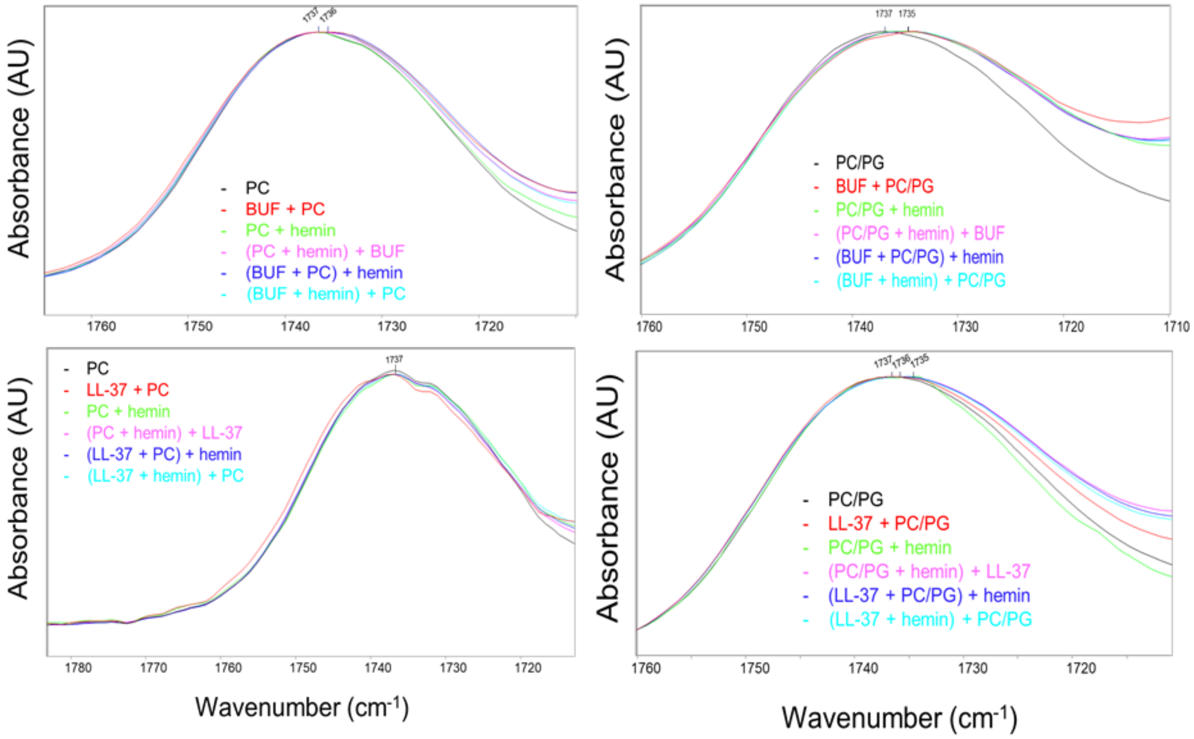

Figure S9. IR analysis on the peptide-induced perturbation of the lipid bilayers: ester carbonyl region. Significant variations observed are listed in the Table below.

Table S4. IR analysis on the peptide-induced perturbation of the lipid bilayers: ester carbonyl region. Significant variations observed for the lipid C=O vibrations in the spectra shown above.

Mixture	Lipid perturbation
BUF + PC	
LL-37 + PC	pep+lip ←
BUF + PC/PG	all →, most pep+lip
LL-37 + PC/PG	all →

← or →, right or red shift of the band maximum compared to the sole lipids

pep, peptide; hem, hemin; lip, lipid/liposome

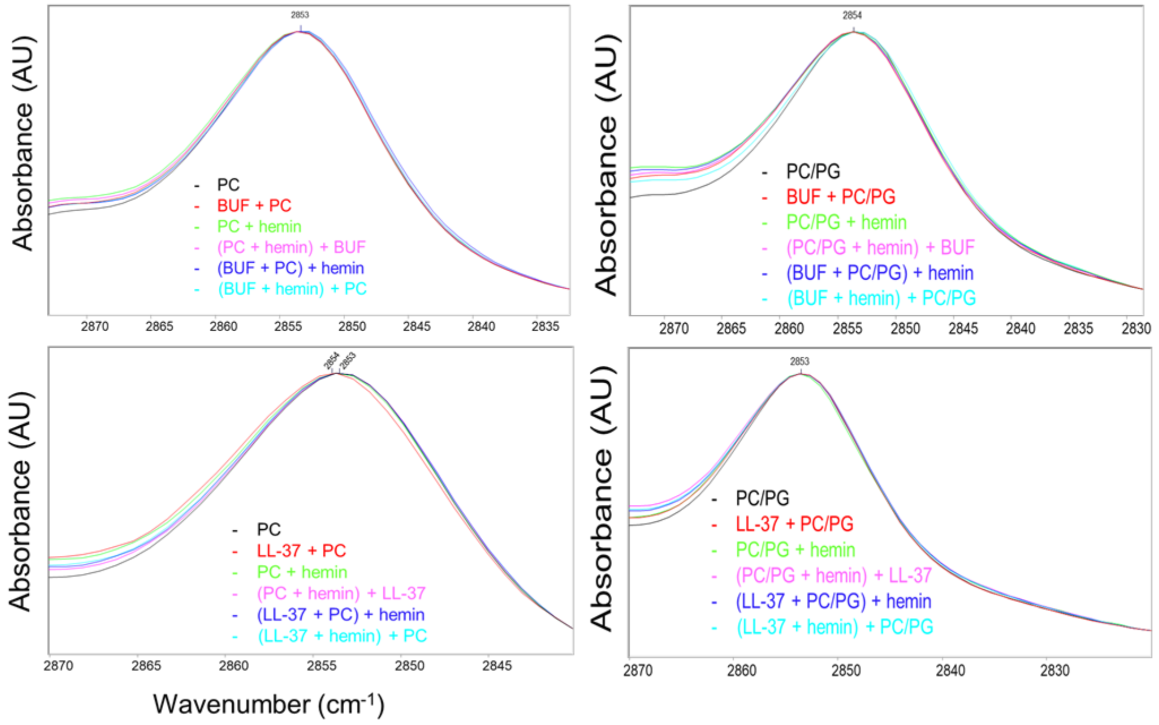

Figure S10. IR analysis on the peptide-induced perturbation of the lipid bilayers: acyl chain region, lipid order and packing. Significant variations observed are listed in the Table below.

Table S5. IR analysis on the peptide-induced perturbation of the lipid bilayers: acyl chain region, lipid order and packing. Significant variations observed for the lipid CH vibrations in the spectra shown above.

Mixture	Lipid perturbation
BUF + PC	(pep+lip)+hem →, (pep+hem)+lip →
LL-37 + PC	pep+lip ←
BUF + PC/PG	all←, except (pep+hem)+lip →
LL-37 + PC/PG	

← or →, right or red shift of the band maximum compared to the sole lipids
pep, peptide; hem, hemin; lip, lipid/liposome

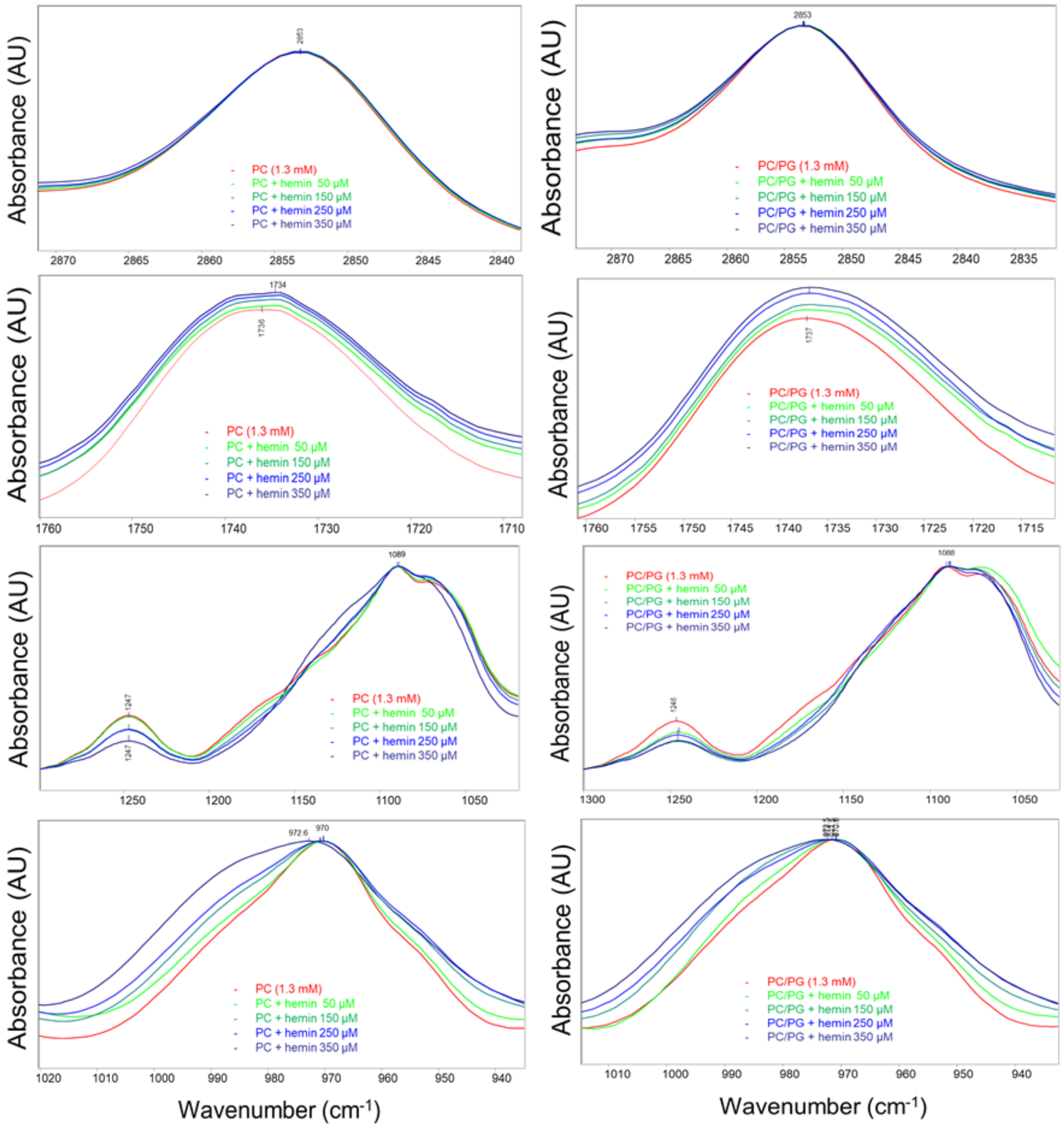

Figure S11. IR analysis: hemin-induced perturbation of the lipid bilayers.

Conclusions derived for the hemin-lipid interaction:

- binding of hemin is detectable for both PC and PC/PG (containing 80% PC)
- hemin makes lipid packing slightly less ordered as indicated by slightly wider bands at 2853 cm^{-1}
- hemin binding down to the lipid neck region is indicated by changing the ratio of the H-bonded (at 1734 cm^{-1}) and non-bonded population (at 1738 cm^{-1}) of the ester carbonyl group [6] resulting in an overall shift of the C=O stretching band envelop, and the effect is observed at all hemin concentrations with PC, however, it is pronounced only at lower hemin levels with PC/PG.
- lipid head-group phosphate is perturbed at all lipid to peptide ratios, the effect is highest at highest hemin ratios with PC, but at lowest hemin levels with PC/PG.
- peptide is associated to lipid head-group cholines of PC at all hemin to peptide ratios with both PC and PC/PG, the effect is dependent on the hemin concentration. This can presumably be attributed to electrostatic attraction between the positively charged choline and the negatively charged carboxyl groups of hemin.

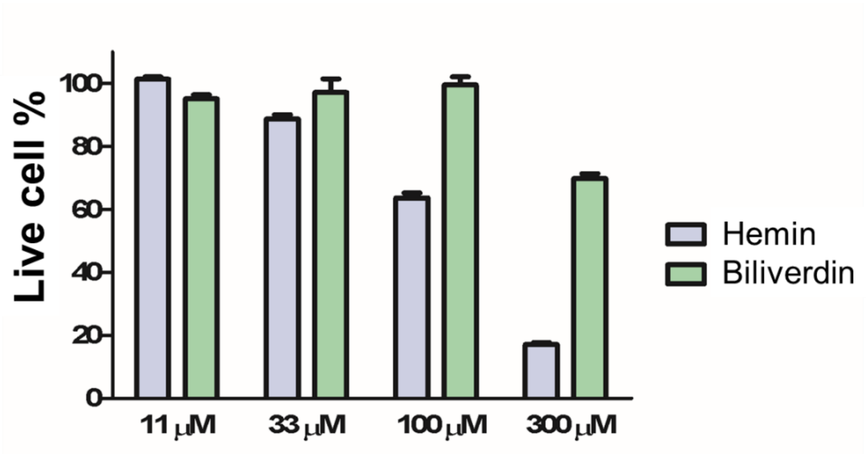

Figure S12. Cytostatic effect of hemin and biliverdin on EBC-1 cells. Viability assays were performed on EBC-1 cells as described in the Methods section.
No significant toxicity was detected up to 100 μM for biliverdin while hemin showed a dose-dependent cytostatic effect at ≥33 μM, with estimated 50% live cells at ~ 140-150 μM.

References

1 Gautier, R., Douguet, D., Antonny, B. & Drin, G. HELIQUEST: a web server to screen sequences with specific alpha-helical properties. *Bioinformatics* **24**, 2101-2102, doi:10.1093/bioinformatics/btn392 (2008).

2 Brauner, J. W., Mendelsohn, R. & Prendergast, F. G. Attenuated total reflectance Fourier transform infrared studies of the interaction of melittin, two fragments of melittin, and delta-hemolysin with phosphatidylcholines. *Biochemistry* **26**, 8151-8158, doi:10.1021/bi00399a020 (1987).

3 Queme-Pena, M. *et al.* Manipulating Active Structure and Function of Cationic Antimicrobial Peptide CM15 with the Polysulfonated Drug Suramin: A Step Closer to in Vivo Complexity. *Chembiochem* **20**, 1578-1590, doi:10.1002/cbic.201800801 (2019).

4 Lee, M. T., Sun, T. L., Hung, W. C. & Huang, H. W. Process of inducing pores in membranes by melittin. *Proc Natl Acad Sci U S A* **110**, 14243-14248, doi:10.1073/pnas.1307010110 (2013).

5 Svensson, F. R., Lincoln, P., Norden, B. & Esbjorner, E. K. Tryptophan orientations in membrane-bound gramicidin and melittin-a comparative linear dichroism study on transmembrane and surface-bound peptides. *Biochim Biophys Acta* **1808**, 219-228, doi:10.1016/j.bbamem.2010.10.004 (2011).

6 Blume, A., Hübner, W. & Messner, G. Fourier transform infrared spectroscopy of 13C: O labeled phospholipids hydrogen bonding to carbonyl groups. *Biochemistry* **27**, 8239-8249 (1988).
